# Supplementary material for: APIR: Aggregating Universal Proteomics Database Search Algorithms for Peptide Identification with FDR Control
Source: Genomics Proteomics Bioinformatics. 2024 Jun 3;22(2):qzae042. doi: 10.1093/gpbjnl/qzae042 (PMC12536914; doi:10.1093/gpbjnl/qzae042)
Supplement: qzae042_Supplementary_Data [file qzae042_supplementary_data.zip › Figure S8 E.pdf]

FDR threshold 1% (Scaffold variant)

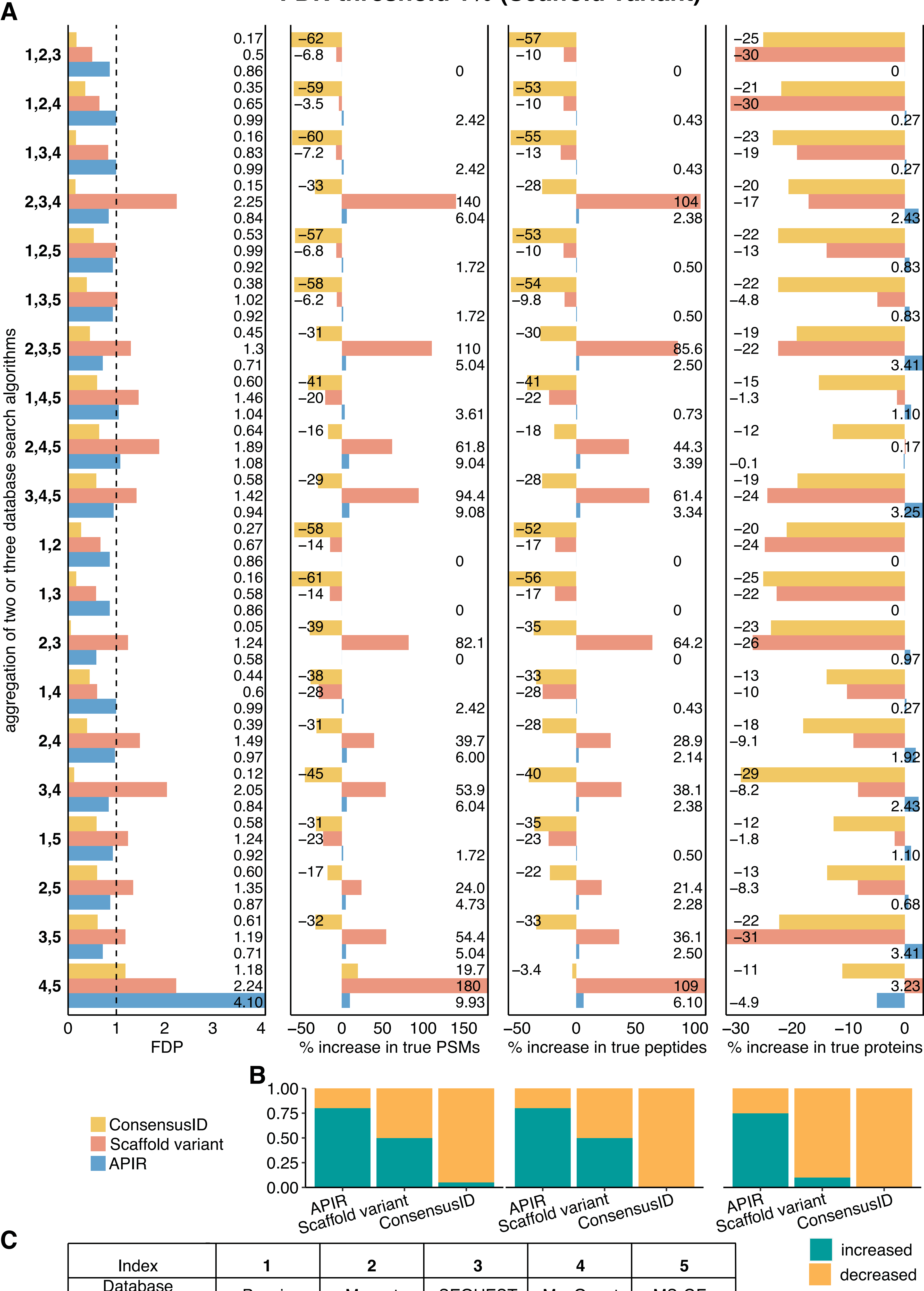

C

Index	1	2	3	4	5
Database search algorithm	Byonic	Mascot	SEQUEST	MaxQuant	MS-GF+
Round 1	q-thre	q-thre	q-thre	APIR-adjust	q-thre
