# Supplementary material for: APIR: Aggregating Universal Proteomics Database Search Algorithms for Peptide Identification with FDR Control
Source: Genomics Proteomics Bioinformatics. 2024 Jun 3;22(2):qzae042. doi: 10.1093/gpbjnl/qzae042 (PMC12536914; doi:10.1093/gpbjnl/qzae042)
Supplement: qzae042_Supplementary_Data [file qzae042_supplementary_data.zip › File S1.docx]

**Supplementary information**

# Phospho-proteomics AML datasets generation

Frozen cell lysates were further diluted with 7.2 M guanidinium hydrochloride (GuHCl) with 100 mM ammonium bicarbonate, reduced with 10 mM dithiothreitol (DTT) for 30 min at 56^◦^C, and alkylated with 22.5 mM iodoacetamide for 30 min protected from light. After diluting GuHCl to a concentration of 1 M, proteins were digested overnight with trypsin at 37^◦^C. Peptides were desalted by C18, dried by vacuum centrifugation, labeled with trimethylthiazoline (TMT) stable isotope labeling reagents (ThermoFisher Scientific, Madison, WI), combined, and dried by vacuum centrifugation. Combined labeled peptides were desalted to remove labeling by-products, and phosphopeptides were enriched by immobilized metal affinity chromatography as described [1]. Enriched phosphopeptides were analyzed by high-pH reversed phase (RP), strong anion exchange (SAX), and low-pH reversed phase (RP) at a depth of 13 fractions as described [1].

# Post-processing

## *Master protein recommendation*

For a given peptide-spectrum match (PSM), database search algorithms may disagree on its protein, causing difficulties in downstream analysis. Aggregation of Peptide Identification Results (APIR) tackles this issue using a majority vote. Specifically, APIR selects the most frequently reported protein across database search algorithms for the given PSM. If there is a tie, APIR outputs all tied proteins.

## *Post-translational modification recommendation*

For a given PSM, how APIR aggregates its modifications across database search algorithms depends on the type of modifications: static or variable. Static modifications occur universally at every instance of a specified amino acid residue or terminus. For example, tandem mass tags occur at every *N*-termimal. Since static modifications are known and could be specified in the database search process, different database search algorithms will agree in terms of the locations and types of static modifications. Therefore, for any PSM, APIR simply outputs its static modifications by any database search algorithm based on user specification. The default static modification used by APIR includes cysteine carbamidomethylation and tandem mass tags at *N*-terminal and lysine.

Unlike static modifications, variable modifications do not apply to all instances of an amino acid residue. For example, phosphorylation typically occurs at only one or few serines in a peptide with many serines. Because variable modifications are hard to detect, database search algorithms may disagree in the types (such as phosphorylation versus oxidation) and/or sites of modifications; however, they always agree on the number of modifications. Suppose database search algorithms report *M* modifications for the given PSM. To handle these potential disagreements, APIR uses one of the two strategies to recommend variable modifications for a given PSM: PhosphoSitePlus (PSP)-free or PSP-based. In a PSP-free modification recommendation, APIR first counts the number of database search algorithms that report each modification — a combination of modification type and site. Then APIR reports the top *M* most frequently reported variable modifications. A PSP-based modification strategy is similar to PSPfree except for the handling of tied phosphorylation sites. When there is a tie among phosphorylation sites, APIR reports the most frequently studied phosphorylation sites by searching the literature hits on PSP ([https://www.phosphosite.org/)](https://www.phosphosite.org/), a manually curated and interactive resource for studying protein modifications. In particular, PSP has cataloged and counted existing literature and experiments by phosphorylation. Based on PSP, APIR reports the modification with the largest number of high-throughput literature hits if there is a tie between phosphorylations. If doing so fails to identify a unique modification, APIR compares their numbers of Cell Signaling Technology mass spectrometry studies that found the given phosphorylation and report the largest-numbered phosphorylation. If this fails to provide a unique modification, APIR will report the ties.

## *Abundance aggregation*

At the PSM level, APIR first averages a PSM’s abundance across database search algorithms. Then APIR performs normalization by scaling *a_ij_*, which denotes the averaged abundance of PSM *i* in channel *j*, by 10^6^*/*(^P^*_i_ a_ij_*) so that resulting normalized samples will have total abundance 10^6^.

To obtain the abundance at the peptide level, APIR averages the abundance of PSMs containing the same peptide and then performs a scaling across channels such that its cross-channel average equals 100. Specifically, let *b_ij_* denotes the averaged abundance of peptide *i* in sample *j*. The normalized abundance would be 100*b_ij_/*(^P^*_j_ b_ij_*).

To obtain the abundance at the protein level, APIR averages the abundance of PSMs with the same recommended master protein and then performs the same row normalization as it does at the peptide level.

# *Simulation studies*

Here we describe how we conducted the simulation studies. Suppose that we have a total of 10^4^ mass spectra and that target PSMs and decoy PSMs are ordered in such a way that the *i*-th target PSM shares the same mass spectrum as the *i*-th decoy PSM. Among the 10^4^ target PSMs, 1500 are true PSMs, and the rest are false. Let *T_ij_* and *D_ij_* denote the matching score of the *i*-th target PSM and the matching score of the *i*-th decoy PSM by toy database search algorithm *j*, *j* = 1*,...,*6. In addition, we generated missing indices M_1_, M_2_, and M_3_ ⊂ {1*,*2*,...,*10^4^} by randomly sampling without replacement 1000, 2000, and 3000 indices from {1*,*2*,...,*10^4^}. We set M*_j_*_+3_ = M*_j_* for *j* = 1*,*2*,*3. We generated 200 simulated datasets under either the shared-true-PSMs scenario or the shared-false-PSMs scenario using the following procedures.

Under the shared-true-PSMs scenario, we generated the target and decoy output of toy search algorithms 1*,*2*,*3 using the following procedure. If the *i*-th target PSM is true, we generated *X_i_* from the exponential distribution with mean 8, *Y_i_* from the exponential distribution with mean 1 and set *T_i_*_1_ =*T_i_*_2_ = *T_i_*_3_ = *X_i_* and *D_i_*_1_ = *D_i_*_2_ = *D_i_*_3_ = *Y_i_*; if the *i*-th target PSM is false, we generated *T_i_*_1_, *T_i_*_2_, *T_i_*_3_, *D_i_*_1_, *D_i_*_2_, *D_i_*_3_ independently from exponential with mean 1. Under the shared-false-PSMs scenario, we generated the target and decoy output of toy search algorithms 4*,*5*,*6 using the following procedure. If the *i*-th target PSM is true, we generated *T_i_*_4_, *T_i_*_5_, *T_i_*_6_ independently from exponential with mean 4 and *D_i_*_4_, *D_i_*_5_, *D_i_*_6_ independently from exponential with mean 1; if the *i*-th target PSM is false, we first generated *X_i_* and *Y_i_* independently from the exponential distribution with mean 1 and then set

*T_i_*_4_ = *T_i_*_5_ = *T_i_*_6_ = *X_i_* and *D_i_*_4_ = *D_i_*_5_ = *D_i_*_6_ = *Y_i_*. Under either scenario, we set *T_ij_* to be a missing value if *i* ∈M*_j_* so that each algorithm captures unique target PSMs.

We examined the actual false discovery rates (FDRs) of APIR-FDR on each toy database search algorithm and of aggregation methods: union or intersection of the identified PSM sets from individual database search algorithms, and APIR at the FDR threshold *q* = 5%. For each FDR-control method, we calculated an false discovery proportion (FDP) — the proportion of identified PSMs that are false — on each simulated data and averaged those 200 FDPs to compute the FDR. To obtain the FDP of APIR-FDR, we applied APIR-FDR with the FDR threshold *q* = 5% to each toy database search algorithm. To obtain the FDP of union/intersection, we took the union/intersection of the three sets of identified target PSMs by APIR-FDR, one per each toy database search algorithm. To obtain the FDP of APIR, we applied the default APIR to aggregate the three toy database search algorithms with the FDR threshold *q* = 5%.

# Implementation of database search algorithms

## *On the proteomics standard*

Byonic, SEQUEST, and Mascot

Byonic, SEQEUST, and Mascot were each run in Proteome Discoverer 2.3.0.523 (Thermo Fisher Scientific, Waltham, MA). The following settings were used for all 5 database search algorithms: 10 ppm precursor tolerance; 0*.*6 Da fragment tolerance; static modifications: methylthio (C); dynamic modifications: deamination (NQ), oxidation (M). Percolator was used in conjunction with both SEQUEST and Mascot, and the target–decoy mode was set to separate. To acquire the total list of identified PSMs, peptides, and proteins, internal FDRs for all database search algorithms were set to 100%.

MaxQuant

MaxQuant was implemented with the following settings: 10 ppm match tolerance; 0*.*6 Da fragment tolerance; static modifications: methylthio (C); dynamic modifications: deamination (NQ), oxidation (M); second peptide search: true. To acquire the total list of identified PSMs, peptides, and proteins, the internal FDR was set to 100%. MaxQuant outputs a posterior error probability (PEP) for each target PSM and decoy PSM.

MS-GF+

MS-GF+ was implemented with the following settings: 10 ppm match tolerance; static modifications: methylthio (C); dynamic modifications: deamination (NQ), oxidation (M). To acquire the total lists of identified PSMs, peptides, and proteins, the internal FDR was set to 100%.

## *On the phospho AML datasets*

Byonic, SEQUEST, and Mascot

The phospho AML spectra were searched with the following settings: 10 ppm precursor tolerance; 0*.*02 Da fragment tolerance; static modifications: TMT6plex (*N*-term, K), carbamindomethyl (C); dynamic modifications: oxidation (M), Phopho (STY).

MaxQuant

MaxQuant was implemented with the following settings. Group-specific parameters: type: reporter ion MS2, isobaric labels 6plex TMT, filter by precursor intensity fractions (PIF, minimal reported PIF 0*.*75); modifications: variable modifications including oxidation (M) and phosphorylation (Y), fixed modification carbamidomethyl (C), maximal number of modifications per peptide 5; instrument: orbitrap with default parameters except that the first search peptide tolerance is set to 10 ppm; digestion: enzyme Trypsin/P, missed cleavage 2. Global parameters: sequences: contaminants FALSE, minimal peptide length 6, maximal peptide mass (Da) 10,000 Da, and the rest parameters are default; advanced identification: use second peptides (default); tandem mass spectrometry (MS/MS), measured in ion trap mass spectrometry(ITMS) (MS/MS-ITMS): all default parameters except that ITMS MS/MS match tolerance 0*.*6 Da; identification: PSM FDR and protein FDR 1, with rest parameters set to default; protein quantification: set “Use only unmodified peptides and...” and “Advanced ratio estimation” to false; tandem mass spectrometry (MS/MS), measured in Fourier transform–based mass spectrometers (FTMS) (MS/MS-FTMS): FTMS MS/MS match tolarance 10 ppm and the rest parameters are set to default. MaxQuant outputs a PEP for each target PSM and decoy PSM.

MS-GF+

MS-GF+ was implemented with the following settings: 10 ppm precursor tolerance; search decoy database: 1 (true); instrument ID: 1 (Orbitrap/FTICR/Lumos); Enzyme ID: 1 (Trypsin); protocol ID: 4(TMT); output additional features: 1 (true); maximum missed cleavages: 2; maximum number of variable modifications per peptide: 4; variable modifications including oxidation (M) and phosphorylation (Y); fixed modification carbamidomethyl (C), TMT6plex (K and *N*-term). To acquire the total lists of identified PSMs, peptides, and proteins, the internal FDR was set to 100%.

## *On the TNBC dataset*

Byonic, SEQUEST, and Mascot

The genistein spectra were searched with the following settings:

20 ppm precursor tolerance; 0*.*02 Da fragment tolerance; static modifications: TMT6plex (*N*-term, K), carbamindomethyl (C); dynamic modififications: oxidation (M), phopho (STY).

MaxQuant

MaxQuant was implemented with the following settings. Group-specific parameters: type: reporter ion MS2, isobaric labels 6plex TMT, filter by PIF (minimal reported PIF 0*.*75); modifications: variable modifications including oxidation (M) and phosphorylation (Y), fixed modification carbamidomethyl (C), maximal number of modifications per peptide 5; instrument: orbitrap with default parameters except that the first search peptide tolerance is set to 20 ppm; digestion: enzyme Trypsin/P, missed cleavage 2. Global parameters: sequences: contaminants FALSE, minimal peptide length 6, maximal peptide mass (Da) 10,000 Da, and the rest parameters are default; advanced identification: use second peptides (default); MS/MS-ITMS: all default parameters except that ITMS MS/MS match tolerance 0*.*6 Da; identification: PSM FDR and protein FDR 1, with rest parameters set to default; protein quantification: set “Use only unmodified peptides and...” and “Advanced ratio estimation” to false; MS/MS FTMS: FTMS MS/MS match tolarance 20 ppm and the rest parameters are set to default. MaxQuant outputs a PEP for each target PSM and decoy PSM.

MS-GF+

MS-GF+ was implemented with the following settings: 20 ppm precursor tolerance; search decoy database: 1 (true); instrument ID: 1 (Orbitrap/FTICR/Lumos); Enzyme ID: 1 (Trypsin); protocol ID: 4(TMT); output additional features: 1 (true); maximum missed cleavages: 2; maximum number of variable modifications per peptide: 4; variable modifications including oxidation (M) and phosphorylation (Y); fixed modification carbamidomethyl (C), TMT6plex (K and *N*-term). To acquire the total lists of identified PSMs, peptides, and proteins, the internal FDR was set to 100%.

## *On the non-phospho AML dataset*

Byonic, SEQUEST, and Mascot

The nonphospho AML spectra were searched with the following settings: 10 ppm precursor tolerance; 0*.*6 Da fragment tolerance; digestion enzyme: Lys-c; static modifications: TMT6plex (*N*-term, K), carbamindomethyl (C); dynamic modifications: oxidation (M).

MaxQuant

MaxQuant was implemented with the following settings. Group-specific parameters: type: reporter ion MS2, isobaric labels 6plex TMT, filter by PIF (minimal reported PIF 0*.*75); modifications: variable modifications oxidation (M), fixed modification carbamidomethyl (C), maximal number of modifications per peptide 5; instrument: orbitrap with default parameters except that the first search peptide tolerance is set to 10 ppm; digestion: enzyme Lysc/P, missed cleavage 2. Global parameters: sequences: contaminants FALSE, minimal peptide length 6, maximal peptide mass (Da) 10000 Da, and the rest parameters are default; advanced identification: use second peptides (default); MS/MS-ITMS: all default parameters except that ITMS MS/MS match tolerance 0*.*6 Da; identification: PSM FDR and protein FDR 1, with rest parameters set to default; protein quantification: set “Use only unmodified peptides and…” and “Advanced ratio estimation” to false; MS/MS FTMS: FTMS MS/MS mat tolerance 20 ppm and the rest parameters are set to default. MaxQuant outputs a PEP for each target PSM and decoy PSM.

MS-GF+

MS-GF+ was implemented with the following settings: 10 ppm precursor tolerance; search decoy database: 1 (true); instrument ID: 1 (Orbitrap/FTICR/Lumos); Enzyme ID: 3 (Lys-C); protocal ID: 4(TMT); output additional features: 1 (true); maximum missed cleavages: 2; maximum number of variable modifications per peptide: 4; variable modifications oxidation (M); fixed modification carbamidomethyl (C), TMT6plex (K and *N*-term). To acquire the total lists of identified PSMs, peptides, and proteins, the internal FDR was set to 100%.

# Existing aggregation methods

## *Implementation of Scaffold*

Scaffold (Proteome Software, Portland, OR) adopts a Bayesian approach to aggregate probabilities of the individual database search algorithm results into a single probability for each PSM. One of its key step is to generate for each database search algorithm a peptide probability model that estimates the probability of an individual spectrum being correctly assigned to a peptide based on that database search algorithm’s score. To realize this, Scaffold designs a different statistical model for the internal scores from each database search algorithm [2], making it difficult to generalize its approach to other database search algorithms. Scaffold supports Byonic (Protein Metrics, Cupertino, CA), Mascot (Matrix Science, Cupertino, CA), Mascot Distiller (Matrix Science, Cupertino, CA), MaxQuant/Andromeda (Max Planck Institute, Stuttgart, Germany), PEAKS (Bioinformatics Solutions Inc, Waterloo, ON, Canada), and Proteome Discoverer (Thermo Fisher Scientific, Waltham, MA) database search algorithms including Byonic, SEQUEST, and Mascot.

We used Scaffold to combine the outputs of Byonic, Mascot, SEQUEST, MaxQuant, and MS-GF+ on the proteomics standard. For each combination of database search algorithms, the result files were inputted into Scaffold Q+ (version 4.10.0, Proteome Software Inc., Portland, OR) to generate peptide and protein identification probabilities. Peptide probabilities were assigned by the Scaffold Local FDR algorithm, protein groups were generated using standard experiment-wide protein grouping, and protein probabilities were assigned by the Protein Prophet algorithm [3]. To compare Scaffold with APIR which aims to control the FDR at the PSM level, we implemented Scaffold in two ways. In the first implementation, we set both the peptide threshold and the protein threshold to be *q* FDR, the FDR threshold of APIR. In the second comparison, we set the peptide threshold to be *q* FDR and varied the protein threshold among all default thresholds: 20% (1 − PEP), 50%, 80%, 90%, 95%, 99%, 99*.*9%, 1% FDR, 2% FDR, 3% FDR, 5% FDR and 10% FDR to maximize the number of identified peptides.

## *Implementation of ConsensusID*

ConsensusID is part of the OpenMS Proteomics Pipeline [4]. It adopts a probabilistic approach to aggregate the top-scoring PSM results from several database search algorithms. A key feature of this tool is its sequence similarity scoring mechanism, which is a method to estimate the matching scores for PSMs in cases when the peptide is missing from the high-ranking results of a database search algorithm. It involves fitting the matching scores from each database search algorithm as a two-component mixture model. The two components are a Gumbel distribution for the incorrect PSMs and a normal distribution for the correct PSMs [5]. Although the paper Nahnsen et al. [5] claimed that ConsensusID supports all database search algorithms, the OpenMS pipeline only supports the following database search algorithms: Comet (open-source: https://comet-ms.sourceforge.net/)[6], CompNovo (open-source: https://carta.tech/man-pages/man1/CompNovo.1.html)[7], Crux (open-source: https://crux.ms/) [8], Mascot (Matrix Science, Cupertino, CA), MS-GF+ (open-source: https://msgfplus.github.io/) [9], MyriMatch (open-source: https://medschool.vanderbilt.edu/msrc-bioinformatics/software) [10], OMSSA (open-source: https://proteomicsresource.washington.edu/protocols06/omssa.php) [11], PepNovo (open-source: https://github.com/jmchilton/pepnovo) [12], and X!Tandem (open-source: https://www.thegpm.org/tandem/) [13].

We used OpenMS (version 2.6.0) to combine the search results of Byonic, Mascot, SEQUEST, MaxQuant, and MS-GF+ on the proteomics standard dataset using the following procedure.

We first converted xlsx output from search engines to idXML files in Python. We then used TOPPAS GUI: the OpenMS proteomics pipeline assistant with the following nodes: input file, merge, IDMerger, consensusID, and output file. Next, we used the default settings of IDMerger. Finally, the settings of consensusID: set “per spectrum” to TRUE, with the rest parameters set to the default.

# DE peptides analysis of the phospho AML-C1 dataset

Here we describe how we performed differential expression (DE) analysis on the phospho AML-C1 dataset. This dataset contains six bone marrow samples: one leukemia stem cells (LSC)-enriched sample and one LSC-depleted sample from patient P5337, two LSC-enriched samples and one LSC-depleted sample from patient P5340, and one control.

Using all six samples from the phospho AML-C1 dataset, we first applied APIR to combine the search results by MaxQuant and MS-GF+. Then we applied APIR to adjust the search results of MaxQuant and MS-GF+ separately. Next, we selected fours sample: the two samples from P5337 and the LSC-depleted sample from P5340 and one of the two LSC-enriched samples from patient P5340. We treated the LSC-enriched samples and the LSC-depleted samples as from two conditions and applied DESeq2 with FDR threshold 5% for DE analysis [14]. We use the R package DESeq2 version 1.28.1.

# The differences between machine-learning based aggregation methods such as PepArML[15] and a statistical framework such as APIR

Machine learning methods such as PepArML [15] use a benchmark proteomics dataset with known true PSMs and false PSMs as the training data to train a classifier. Then given a new proteomics dataset, users could apply the trained classifier to predict whether a target PSM is true or false. While these methods do utilize more information, it relies on a highly arguable assumption that the information learned from the benchmark dataset is applied to the new proteomics dataset.

Accordingly, the major advantage of these machine learning methods is that when the information from the training data is applied, the additional information from the training data is likely to improve the power of peptide identification. The major drawback of these methods is that when the training data is different from the new proteomics data (which is often the case given the well-recognized diversity in biological samples), the classifier learned on the training data could produce misleading results.

In contrast, the major advantage of a statistical framework is that it has interpretability. The drawback of a statistical framework that does not rely on training data is that it could not borrow information from external sources.

**Why the numbers of true PSMs keep unchanged as the FDR increased from** 1% **to** 10% **for APIR-FDR compared with MaxQuant?**

When we applied APIR-FDR to the output by MaxQuant, we used −log_10_-transformed PEP output by MaxQuant as the matching scores. Because the target coverage proportion (defined as the proportion of target PSMs whose mass spectra also appear among the decoy PSMs; see Method) is low, APIRFDR uses the *P* value-based approach to control the FDR. Specifically, the *P* value-based approach first computes a *P* value for each target PSM as the proportion of decoy PSMs whose matching scores are no less than the target PSM’s matching score. Then it applies the Benjamini-Hochberg procedure to all target PSMs’ *P* values to achieve the control FDR control.

Figure S3 shows the histogram (gray bars) of the decoy PSMs’ matching scores, whose frequencies have a sharp change at the dashed vertical line. It also shows the relationship (red dots) between the target −log_10_(FDR) (Y-axis) and the matching score threshold (X-axis). When the matching score threshold is at the dashed vertical line, there is a sudden change in the corresponding *P* value threshold (and thus the FDR threshold, which changes from 1% to 10%) because of the sharp frequency change of decoy PSMs’ matching scores at this threshold. This explains why APIR-FDR identifies the same set of PSMs at this matching score threshold, but the FDR threshold changes from 1% to 10%.

In contrast, MaxQuant does not use this *P* value-based approach but calls the target PSMs. with PEPs no greater than the FDR threshold as discoveries. Hence, as the FDR threshold increases, MaxQuants increases the PEP threshold and identifies more PSMs. However, we found that MaxQuant’s PEP thresholding approach failed to control the FDR in Figure 1.

**References**

[1] Wang LD, Ficarro SB, Hutchinson JN, Csepanyi-Komi R, Nguyen PT, Wisniewski E, et al. Phosphoproteomic profiling of mouse primary HSPCs reveals new regulators of HSPC mobilization. Blood 2016;128:1465–74.

[2] Searle BC, Turner M, Nesvizhskii AI. Improving sensitivity by probabilistically combining results from multiple MS/MS search methodologies. J Proteome Res 2008;7:245–53.

[3] Nesvizhskii AI, Keller A, Kolker E, Aebersold R. A statistical model for identifying proteins by tandem mass spectrometry. Anal Chem 2003;75:4646–58.

[4] Rst HL, Sachsenberg T, Aiche S, Bielow C, Weisser H, Aicheler F, et al. OpenMS: a flexible open-source software platform for mass spectrometry data analysis. Nat Methods 2016;13:741–8.

[5] Nahnsen S, Bertsch A, Rahnenfuhrer J, Nordheim A, Kohlbacher O. Probabilistic consensus scoring improves tandem mass spectrometry peptide identification. J Proteome Res 2011;10:3332–43.

[6] Eng JK, Jahan TA, Hoopmann MR. Comet: an open-source MS/MS sequence database search tool. Proteomics 2013;13:22–4.

[7] Bertsch A, Leinenbach A, Pervukhin A, Lubeck M, Hartmer R, Baessmann C, et al. *De novo* peptide sequencing by tandem MS using complementary CID and electron transfer dissociation. Electrophoresis 2009;30:3736–47.

[8] Park CY, Klammer AA, Kall L, MacCoss MJ, Noble WS. Rapid and accurate peptide identification from tandem mass spectra.J Proteome Res 2008;7:3022–7.

[9] Kim S, Pevzner PA. MS-GF+ makes progress towards a universal database search tool for proteomics. Nature Commun 2014;5:5277.

[10] Tabb DL, Fernando CG, Chambers MC. MyriMatch: highly accurate tandem mass spectral peptide identification by multivariate hypergeometric analysis. J Proteome Res 2007;6:654–61.

[11] Geer LY, Markey SP, Kowalak JA, Wagner L, Xu M, Maynard DM, et al. Open mass spectrometry search algorithm. J Proteome Res 2004;3:958–64.

[12] Frank A, Pevzner P. PepNovo: *de novo* peptide sequencing via probabilistic network modeling. Anal Chem 2005;77:964–73.

[13] Craig R, Beavis RC. TANDEM: matching proteins with tandem mass spectra. Bioinformatics 2004;20:1466–7.

[14] Love MI, Huber W, Anders S. Moderated estimation of fold change and dispersion for RNA-seq data with DESeq2. Genome Biol 2014;15:550.

[15] Edwards N, Wu X, Tseng CW. An unsupervised, model-free, machine-learning combiner for peptide identifications from tandem mass spectra. Clin Proteomics 2009;5:23–36.
