# Supplementary material for: APIR: Aggregating Universal Proteomics Database Search Algorithms for Peptide Identification with FDR Control
Source: Genomics Proteomics Bioinformatics. 2024 Jun 3;22(2):qzae042. doi: 10.1093/gpbjnl/qzae042 (PMC12536914; doi:10.1093/gpbjnl/qzae042)

TIF1alpha
LKpSIEER

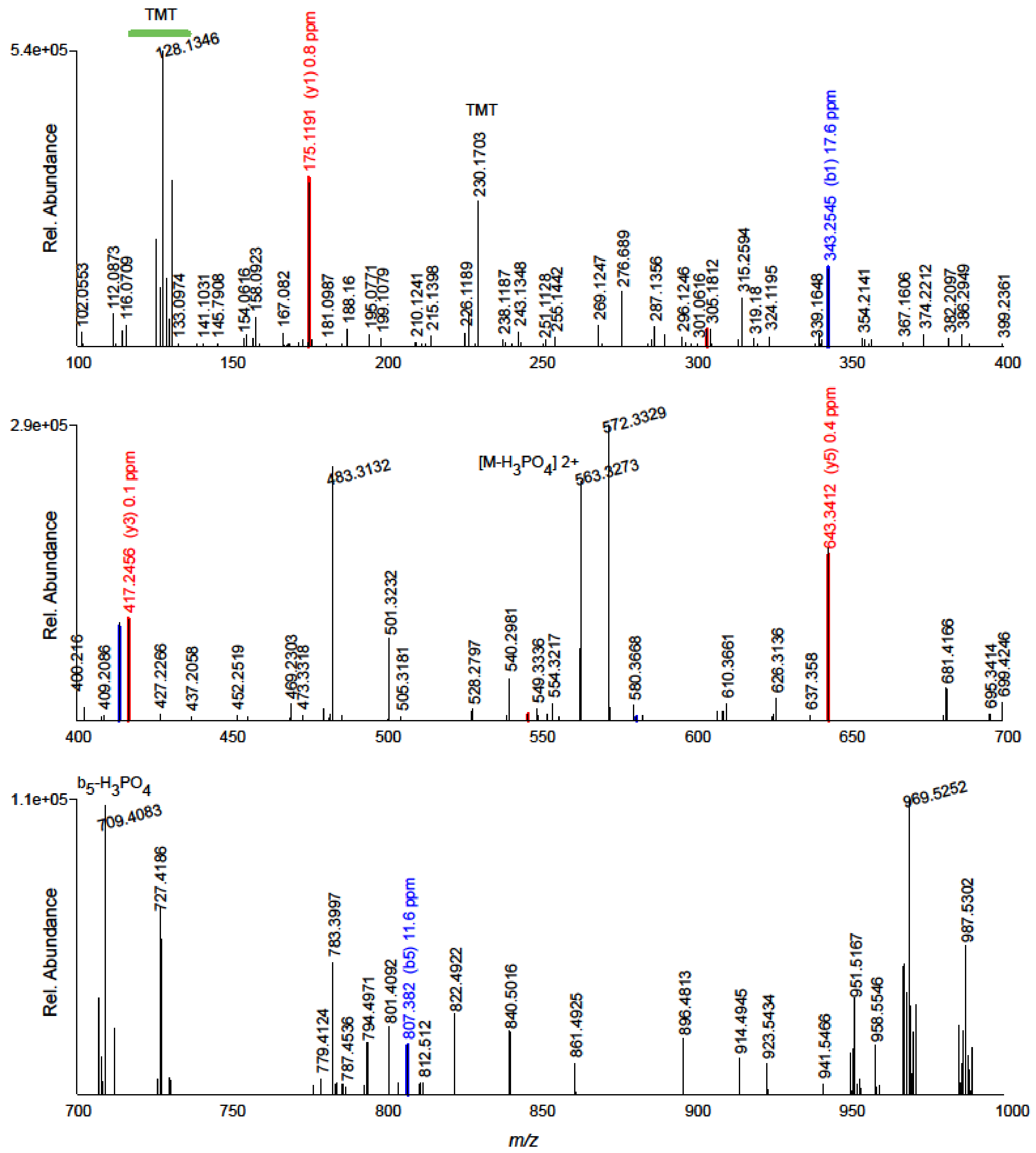

Scan: 33617
RT: 135.97
ms2
FTMS
+prof
612.32
hcd
35.00% NCE
[100.00-1265.00]
Inj: 100.0

PIB5PA
SPGLARFPGLALR

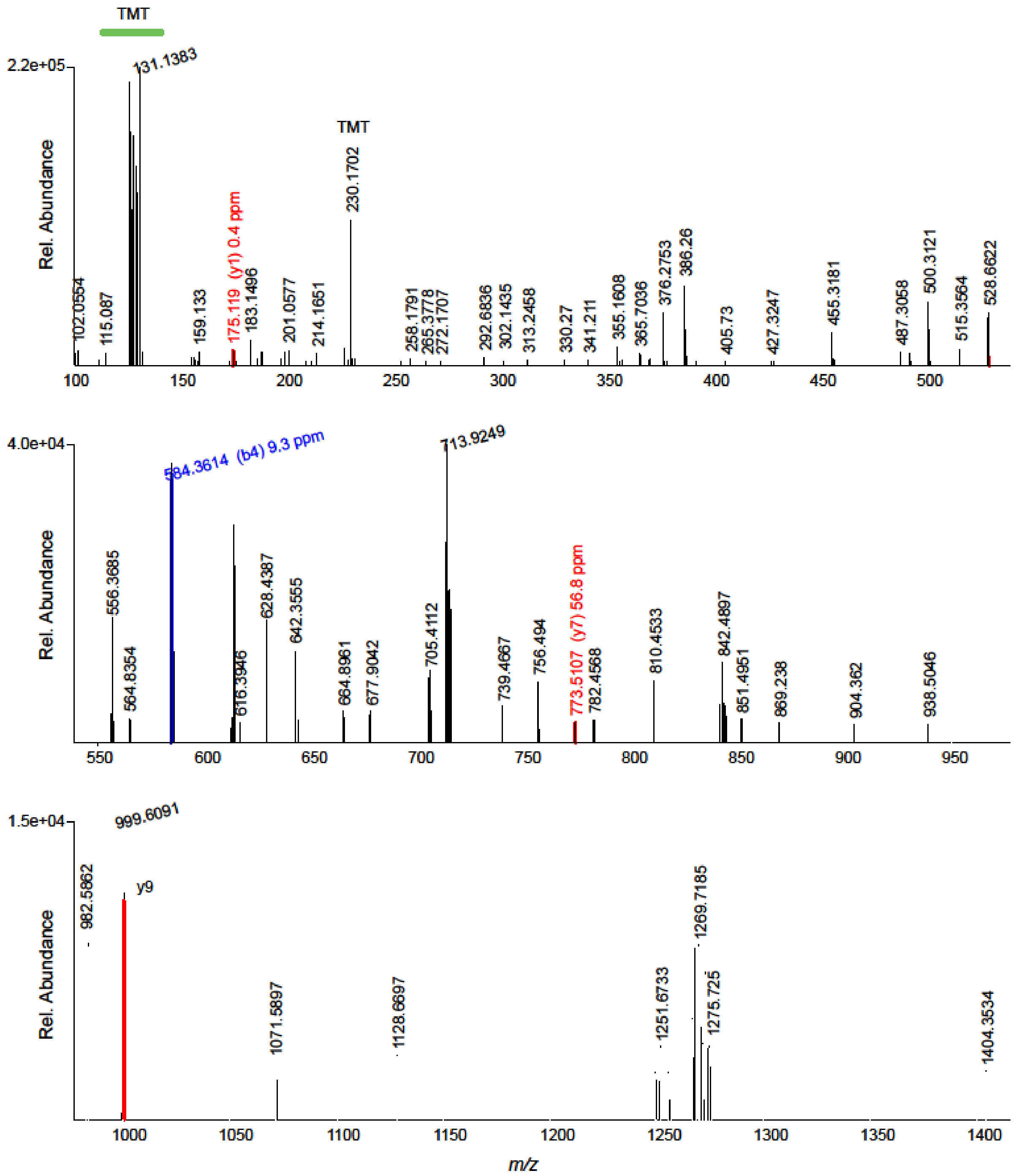

Scan: 24998
RT: 108.23
ms2
FTMS
+prof
528.65
hcd
35.00% NCE
[100.00-1635.00]
Inj: 100.0

HOXB5

IEIAHALCLpSER

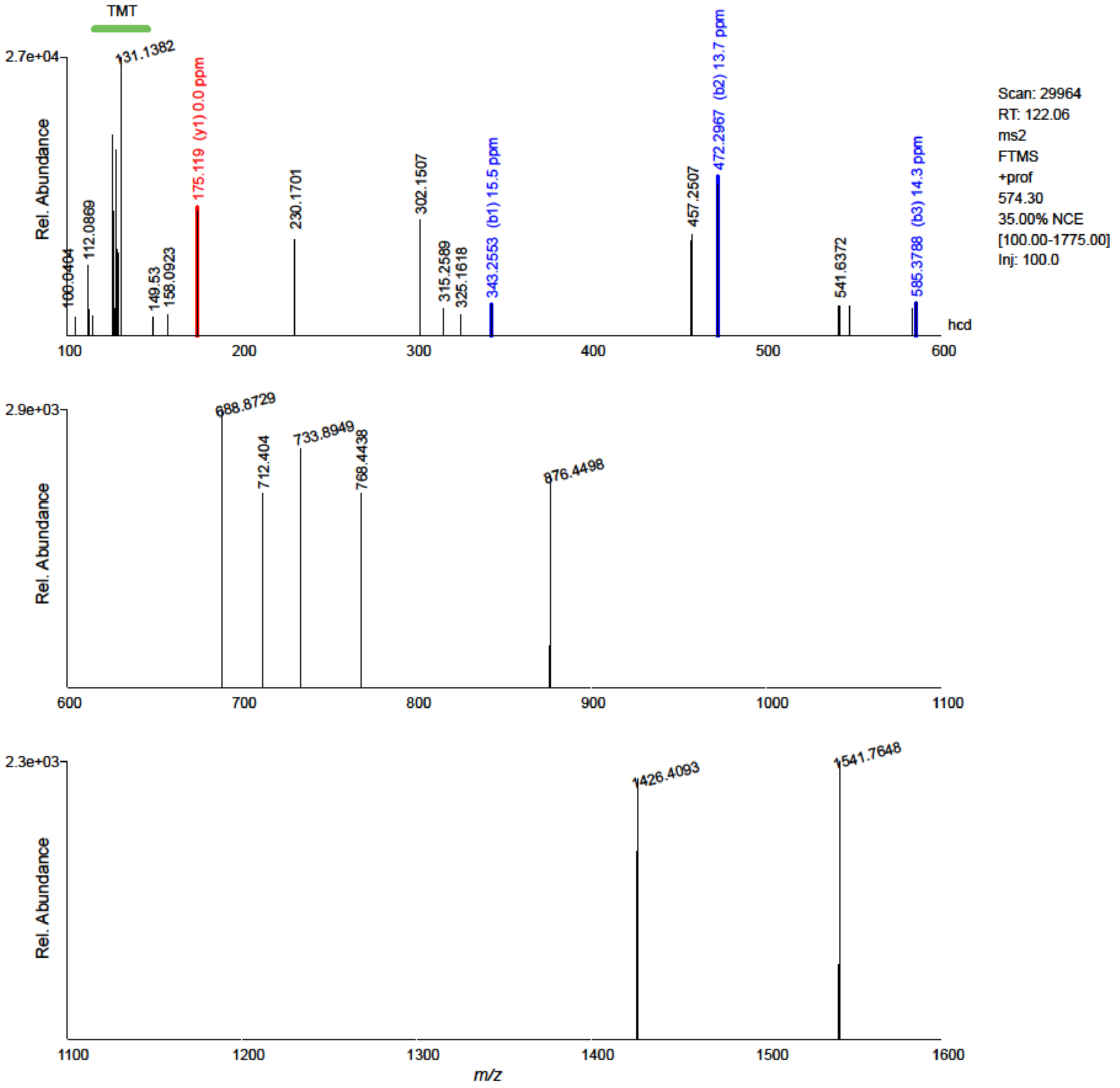

SUMO2
VAGQDGpSVVQFK

Scan: 37871
RT: 147.69
ms2
FTMS
+prof
886.97
hcd
35.00% NCE
[100.00-1830.00]
Inj: 100.0

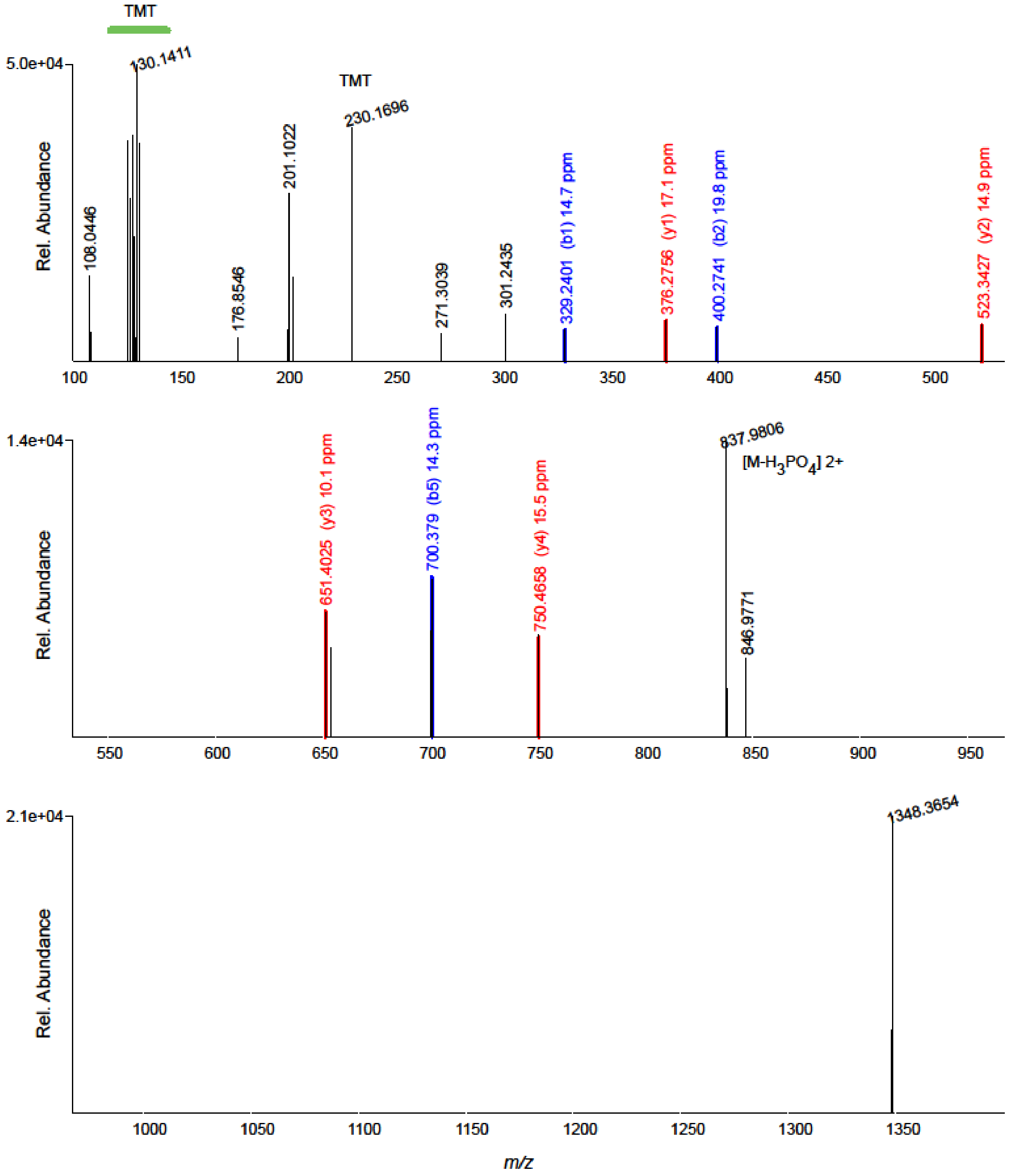

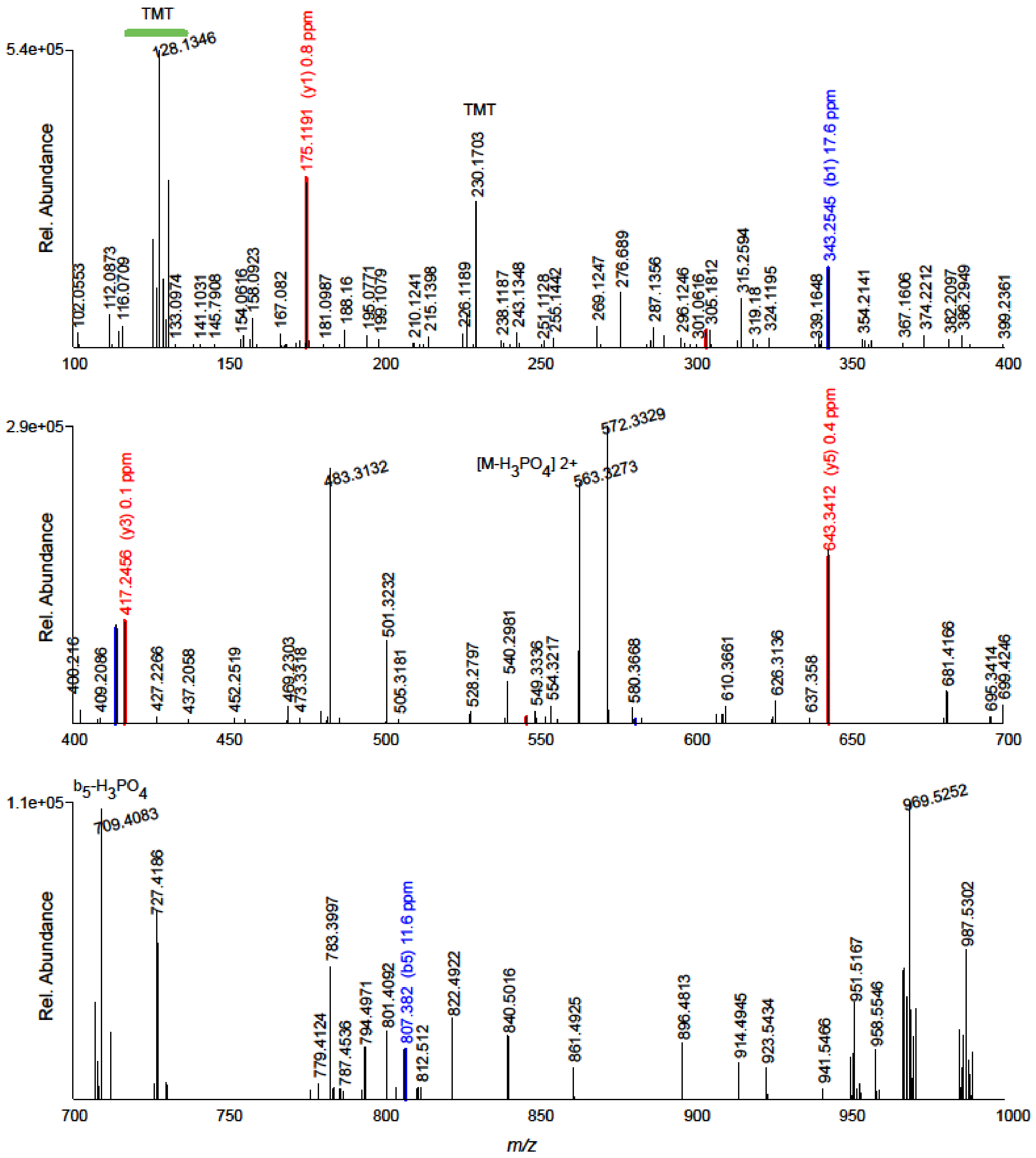

Scan: 33617
RT: 135.97
ms2
FTMS
+prof
612.32
hcd
35.00% NCE
[100.00-1265.00]
Inj: 100.0

JUND

LApSPELER

GPC2
GFWARLSLTVCGDSR

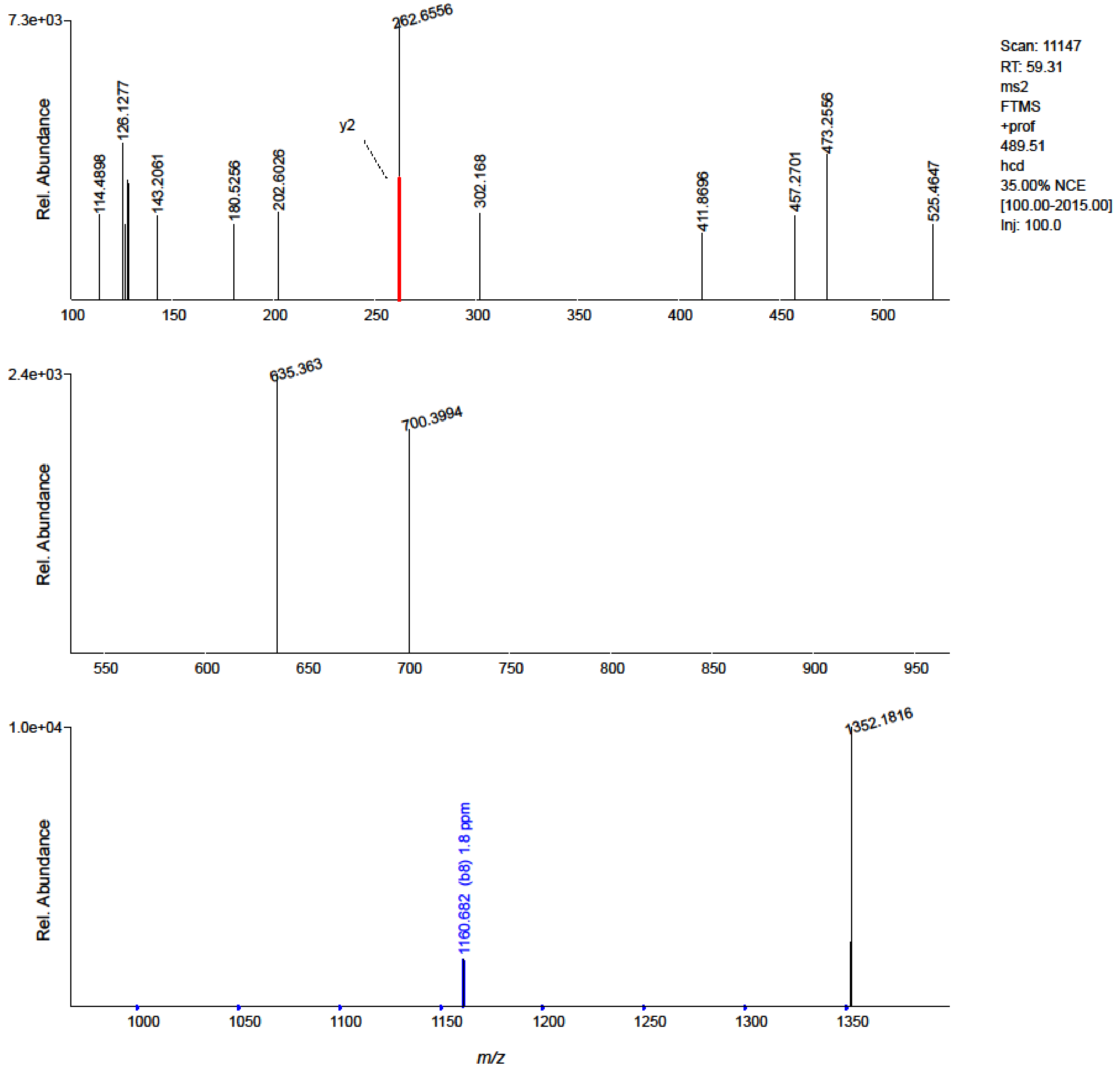

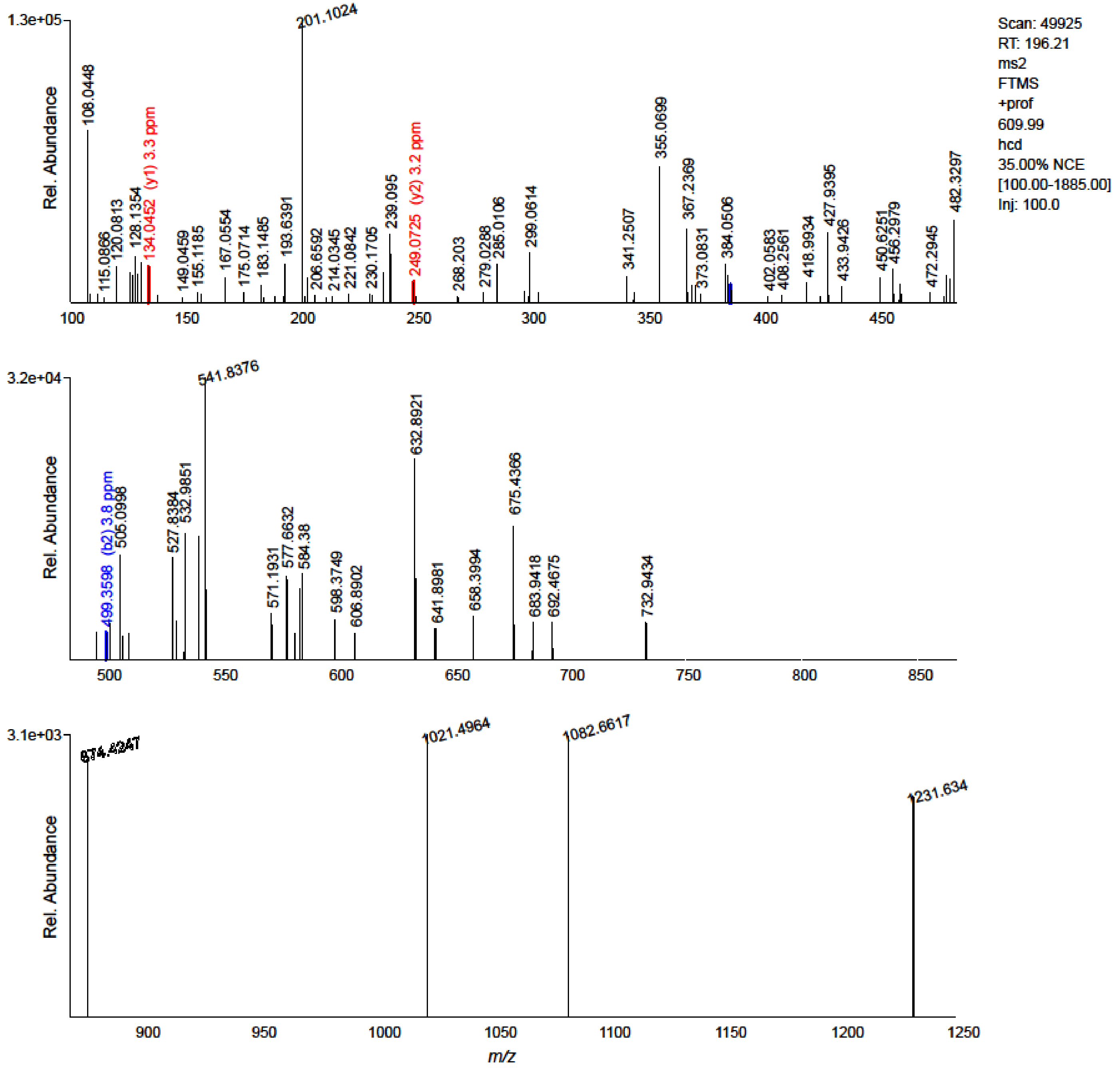

ZFP36L2
RLPIFSRLSISDD

MPO
TITGMCNNRR

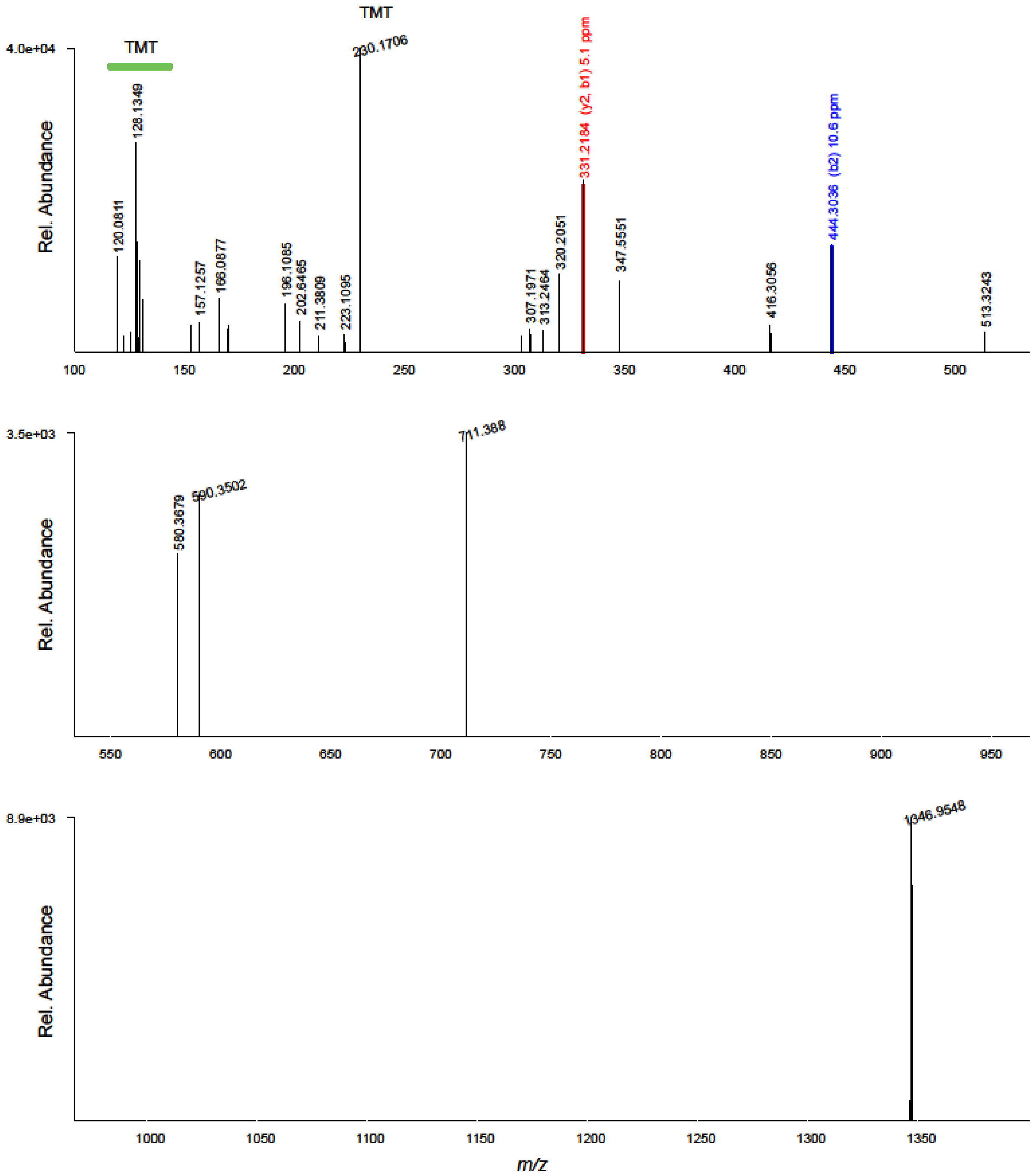

Scan: 26565
RT: 116.22
ms2
FTMS
+prof
484.59
hcd
35.00% NCE
[100.00-1500.00]
Inj: 100.0

JUND
LApSPELER

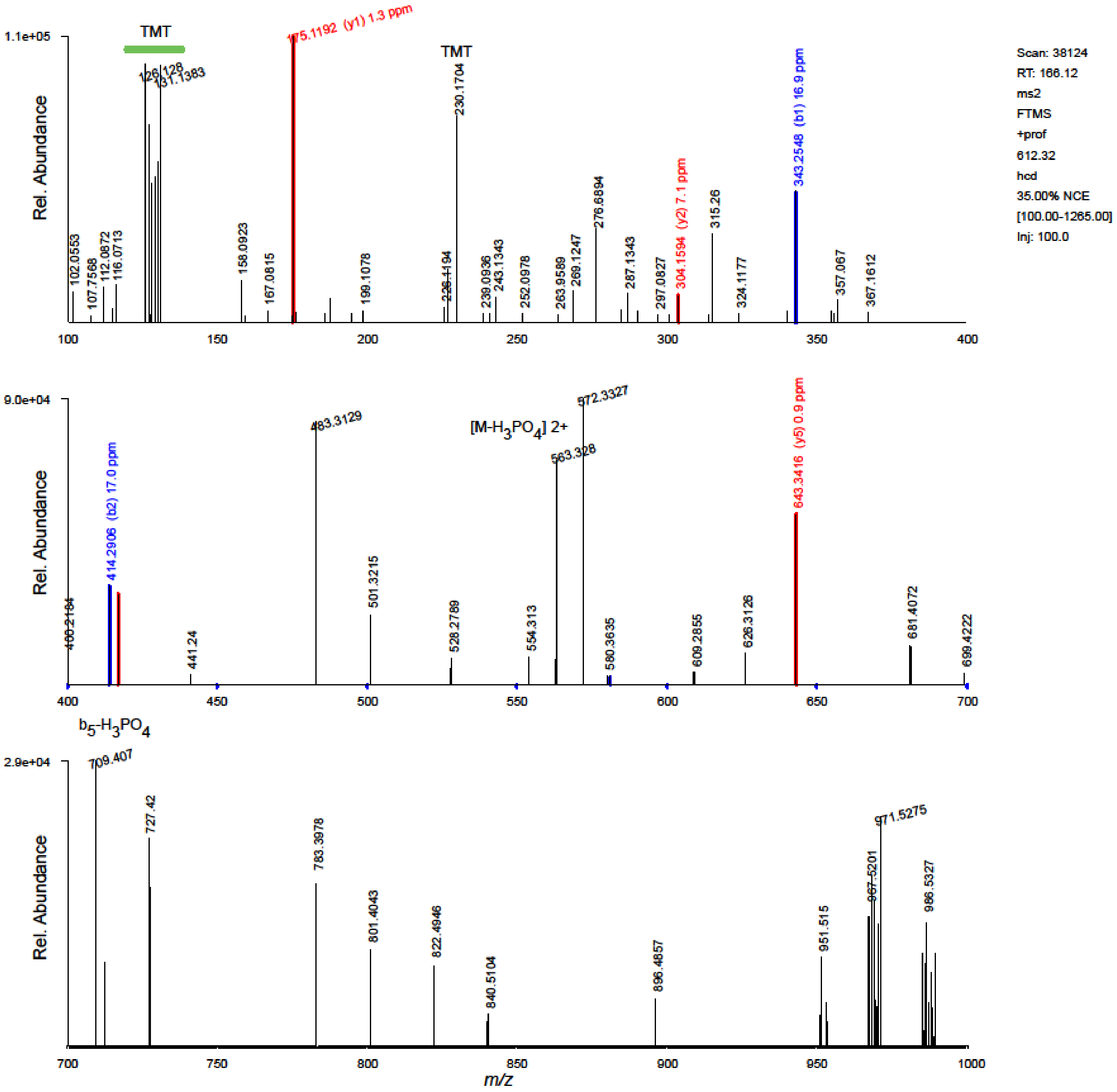

Supplement: qzae042_Supplementary_Data [file qzae042_supplementary_data.zip › File S2.pdf]
