# Supplementary material for: APIR: Aggregating Universal Proteomics Database Search Algorithms for Peptide Identification with FDR Control
Source: Genomics Proteomics Bioinformatics. 2024 Jun 3;22(2):qzae042. doi: 10.1093/gpbjnl/qzae042 (PMC12536914; doi:10.1093/gpbjnl/qzae042)
Supplement: qzae042_Supplementary_Data [file qzae042_supplementary_data.zip › Supplementary material captions.docx]

**Supplementary material**

**File S1 Supplementary information**

**File S2 MS/MS spectra annotation**

**Figure S1 Two implementations of the target–decoy search strategy: concatenated (A) and parallel (B)**

In the concatenated search, a concatenated protein database is created by pooling original protein sequences, called “target” sequences, with the decoy sequences; then a database search algorithm uses the concatenated protein database to find PSMs; consequently, each mass spectra is mapped to either a target sequence or a decoy sequence with only one matching score. In the parallel search, a database search algorithm conducts two parallel searches: a target search where each mass spectrum is matched to target sequences and a decoy search where the mass spectrum is matched to decoy sequences; consequently, each mass spectrum receives two matching scores from the two searches. In both implementations, a PSM is called a target PSM or simply a PSM if it contains a target sequence; otherwise, it is called a decoy PSM.

**Figure S2 Overlaps of true PSMs identified by Byonic, Mascot, and SEQUEST**

Venn diagrams of true PSMs identified by the three database search algorithms from Proteome Discoverer™ software — Byonic, Mascot, and SEQUEST — under the FDR threshold $q=1\%$ (left) or $q=5\%$ (right) on the CPS dataset. The true PSMs identified by Byonic nearly cover the true PSMs identified by Mascot or SEQUEST.

**Figure S3 The change of** $\boldsymbol{-}\mathbf{log}_{\boldsymbol{10}}$**-transformed FDR estimate by APIR (Y-axis) with respect to the matching scores of target PSMs by MaxQuant (X-axis) on the CPS dataset**

We used $-\log_{10}$-transformed PEP output by MaxQuant as the matching scores. The underlying histogram represents the distribution of matching scores of the decoy PSMs by MaxQuant.

**Figure S4 A counterexample explaining why *P* value-free FDR control procedure no longer guarantees to control the FDR after a subset of PSMs is removed**

Suppose that before removing PSMs, at the FDR threshold $10\%$, a database search algorithm reports $10$ PSMs, whose estimated FDRs (q-values or PEPs) are under $10\%$, as discoveries. Among these $10$ PSMs, $1$ is false (in orange), and $9$ are true (in blue), so the actual FDP is $10\%$. In contrast, suppose that we first remove the $5$ PSMs with the smallest q-values or PEPs (crossed out) and next threshold the remaining PSMs at the q-value or PEP threshold $10\%$. Then, the actual FDP becomes 1/5 = 20%.

**Figure S5 Comparing APIR-FDR options on incomplete output from database search algorithms**

At the FDR threshold $q\in\{1\%, \ldots, 10\%\}$, FDPs and power of each of the five database search algorithms when the $1416$ target PSMs (identified by all five database search algorithms at the FDR threshold $q=5\%$) are removed from the output of database search algorithms.

**Figure S6 Comparison of APIR, Scaffold, and ConsensusID on the CPS dataset at the FDR threshold** $\boldsymbol{q=1\%}$ **in terms of FDR control and power**

We set both the peptide threshold and the protein threshold of Scaffold to be 1% FDR. **A.** FDPs (first column), the percentage increases in true PSMs (second column), the percentage increases in true peptides (third column), and the percentage increases in true proteins (fourth column) after aggregating two or three database search algorithms out of the five (Byonic, Mascot, SEQUEST, MaxQuant, and MS-GF+). The percentage increase in true PSMs/peptides/proteins is computed by treating as the baseline the maximal number of correctly identified PSMs/peptides/proteins by individual database search algorithms in round 1 of APIR. **B.** Proportions of combinations that show a non-negative percentage increase (green bars) in true PSMs (first column), true peptides (second column), and true proteins (third column). **C.** The indices of database search algorithms in (A) and the implementation of APIR in round 1. Based on the benchmarking results in Figure 1C, in round 1 of APIR, we applied *P* value-free APIR-FDR to Byonic, Mascot, SEQUEST, and MS-GF+, and we applied *P* value-based APIR-FDR to MaxQuant. In later rounds of APIR, we used *P* value-based APIR-FDR for FDR control.

**Figure S7 Comparison of APIR, Scaffold variant, and ConsensusID on the CPS dataset at the FDR threshold** $\boldsymbol{q=5\%}$ **in terms of FDR control and power**

We set Scaffold’s peptide threshold to be $5\%$ FDR and varied its protein threshold to find the maximal number of identified peptides. **A.** FDPs (first column), the percentage increases in true PSMs (second column), the percentage increases in true peptides (third column), and the percentage increases in true proteins (fourth column) after aggregating two or three database search algorithms out of the five (Byonic, Mascot, SEQUEST, MaxQuant, and MS-GF+). The percentage increase in true PSMs/peptides/proteins is computed by treating as the baseline the maximal number of correctly identified PSMs/peptides/proteins by individual database search algorithms in round 1 of APIR. **B.** Proportions of combinations that show a non-negative percentage increase (green bars) in true PSMs (first column), true peptides (second column), and true proteins (third column). **C.** The indices of database search algorithms in (A) and the implementation of APIR in round 1. Based on the benchmarking results in Figure 1C, in round 1 of APIR, we applied *P* value-free APIR-FDR to Byonic, Mascot, SEQUEST, and MS-GF+, and we applied *P* value-based APIR-FDR to MaxQuant. In later rounds of APIR, we used *P* value-based APIR-FDR for FDR control.

**Figure S8 Comparison of APIR, Scaffold variant, and ConsensusID on the CPS dataset at the FDR threshold** $\boldsymbol{q=1\%}$ **in terms of FDR control and power**

We set Scaffold’s peptide threshold to be $1\%$ FDR and varied its protein threshold to find the maximal number of identified peptides. **A.** FDPs (first column), the percentage increases in true PSMs (second column), the percentage increases in true peptides (third column), and the percentage increases in true proteins (fourth column) after aggregating two or three database search algorithms out of the five (Byonic, Mascot, SEQUEST, MaxQuant, and MS-GF+). The percentage increase in true PSMs/peptides/proteins is computed by treating as the baseline the maximal number of correctly identified PSMs/peptides/proteins by individual database search algorithms in round 1 of APIR. **B.** Proportions of combinations that show a non-negative percentage increase (green bars) in true PSMs (first column), true peptides (second column), and true proteins (third column). **C.** The indices of database search algorithms in (A) and the implementation of APIR in round 1. Based on the benchmarking results in Figure 1C, in round 1 of APIR, we applied *P* value-free APIR-FDR to Byonic, Mascot, SEQUEST, and MS-GF+, and we applied *P* value-based APIR-FDR to MaxQuant. In later rounds of APIR, we used *P* value-based APIR-FDR for FDR control.

**Figure S9 Comparison of APIR and individual database search algorithms on the CPS dataset at the FDR threshold** $\boldsymbol{q=5\%}$ **in terms of FDR control and power**

FDPs (first column), the percentage increases in true PSMs (second column), the percentage increases in true peptides (third column), and the percentage increases in true proteins (fourth column) after aggregating four or five database search algorithms out of the five (Byonic, Mascot, SEQUEST, MaxQuant, and MS-GF+). The percentage increase in true PSMs/peptides/proteins is computed by treating as the baseline the maximal number of correctly identified PSMs/peptides/proteins by individual database search algorithms in round 1 of APIR.

**Figure S10 Comparison of APIR and individual database search algorithms on the CPS dataset at the FDR threshold** $\boldsymbol{q=1\%}$ **in terms of FDR control and power**

FDPs (first column), the percentage increases in true PSMs (second column), the percentage increases in true peptides (third column), and the percentage increases in true proteins (fourth column) after aggregating four or five database search algorithms out of the five (Byonic, Mascot, SEQUEST, MaxQuant, and MS-GF+). The percentage increase in true PSMs/peptides/proteins is computed by treating as the baseline the maximal number of correctly identified PSMs/peptides/proteins by individual database search algorithms in round 1 of APIR.

**Figure S11 The FDPs of peptides identified by APIR and by individual database search algorithms on the *Pfu* CPS dataset at the FDR thresholds** $\boldsymbol{q=1\%}$ **(left) and** $\boldsymbol{q=5\%}$ **(right)**

Note that APIR only aims to control the FDR at the PSM level, not at the peptide level. Since MaxQuant on its own has a high peptide-level FDP, the algorithm combinations that involve MaxQuant are highlighted in red.

**Figure S12 Power improvement of APIR over individual database search algorithms at the FDR threshold** $\boldsymbol{q=1\%}$

The percentage increases in PSMs (first column), the percentage increases in peptides (second column), the percentage increases in peptides with modifications (third column), and the percentage increases in true proteins (fourth column) of APIR after aggregating two or three database search algorithms out of the five (Byonic, Mascot, SEQUEST, MaxQuant, and MS-GF+) at the FDR threshold $q=1\%$ on (**A**) the phospho AML-C1 dataset, (**B**) the phospho AML-C2 dataset, (**C**) the TNBC dataset, and (**D**) the nonphospho AML dataset. The percentage increase in PSMs/peptides/peptides with modifications/proteins is computed by treating as the baseline the maximal number of PSMs/peptides/peptides and modifications/proteins by an individual database search algorithm in round 1 of APIR.

**Figure S13 Power improvement of APIR over individual database search algorithms at the FDR threshold** $\boldsymbol{q=5\%}$

The percentage increases in PSMs (first column), the percentage increases in peptides (second column), the percentage increases in peptides with modifications (third column), and the percentage increases in true proteins (fourth column) of APIR after aggregating four or five database search algorithms out of the five (Byonic, Mascot, SEQUEST, MaxQuant, and MS-GF+) at the FDR threshold $q=5\%$ on (**A**) the phospho AML-C1 dataset, (**B**) the phospho AML-C2 dataset, (**C**) the TNBC dataset, and (**D**) the nonphospho AML dataset. The percentage increase in PSMs/peptides/peptides with modifications/proteins is computed by treating as the baseline the maximal number of PSMs/peptides/peptides and modifications/proteins by an individual database search algorithm in round 1 of APIR.

**Figure S14 Power improvement of APIR over individual database search algorithms at the FDR threshold** $\boldsymbol{q=1\%}$

The percentage increases in PSMs (first column), the percentage increases in peptides (second column), the percentage increases in peptides with modifications (third column), and the percentage increases in true proteins (fourth column) of APIR after aggregating four or five database search algorithms out of the five (Byonic, Mascot, SEQUEST, MaxQuant, and MS-GF+) at the FDR threshold $q=1\%$ on (**A**) the phospho AML-C1 dataset, (**B**) the phospho AML-C2 dataset, (**C**) the TNBC dataset, and (**D**) the nonphospho AML dataset. The percentage increase in PSMs/peptides/peptides with modifications/proteins is computed by treating as the baseline the maximal number of PSMs/peptides/peptides and modifications/proteins by an individual database search algorithm in round 1 of APIR.

**Figure S15 Venn diagrams of identified PSMs by MaxQuant and MS-GF+_on the four real datasets at the FDR threshold** $\boldsymbol{q=1\%}$ **(left) and** $\boldsymbol{q=5\%}$ **(right)**

**Table S1** **The APIR-FDR option of the five search algorithms applied on the CPS dataset and other datasets (Phspho AML-C1, Phspho AML-C2, TNBC, and Nonphospho)**

**Table S2 Evaluation of tandem MS spectra rescued by APIR on phospho AML-C1 and phospho AML-C2**

**Table S3 A list of PSMs with peptides from additional proteins identified by APIR on phospho AML-C1**

**Table S4 A list of PSMs with peptides from additional proteins identified by APIR on phospho AML-C2**

**Table S5 A list of PSMs with peptides from additional proteins identified by APIR on TNBC**
