# Supplementary material for: APIR: Aggregating Universal Proteomics Database Search Algorithms for Peptide Identification with FDR Control
Source: Genomics Proteomics Bioinformatics. 2024 Jun 3;22(2):qzae042. doi: 10.1093/gpbjnl/qzae042 (PMC12536914; doi:10.1093/gpbjnl/qzae042)
Supplement: qzae042_Supplementary_Data [file qzae042_supplementary_data.zip › Table S1_new.docx]

**Table S1 The APIR-FDR option of the five search algorithms applied on the proteomics standard dataset and other datasets (Phspho AML-C1, Phspho AML-C2, TNBC, and Nonphospho)**

| **Dataset** | **Byonic** | **Mascot** | **SEQUEST** | **MaxQuant** | **MS-GF+** |
| --- | --- | --- | --- | --- | --- |
| Standard (default) | *P* value-free | *P* value-based | *P* value-based | *P* value-free | *P* value-free |
| Standard (alternative) | *P* value-based | Clipper | Clipper | *P* value-based | *P* value-based |
| Other datasets (first round) | *P* value-free | *P* value-free | *P* value-free | *P* value-based | *P* value-based |
| Other datasets (other rounds) | *P* value-based | *P* value-based | *P* value-based | *P* value-based | *P* value-based |
