# Supplementary material for: APIR: Aggregating Universal Proteomics Database Search Algorithms for Peptide Identification with FDR Control
Source: Genomics Proteomics Bioinformatics. 2024 Jun 3;22(2):qzae042. doi: 10.1093/gpbjnl/qzae042 (PMC12536914; doi:10.1093/gpbjnl/qzae042)
Supplement: qzae042_Supplementary_Data [file qzae042_supplementary_data.zip › Figure S1 E.pdf]

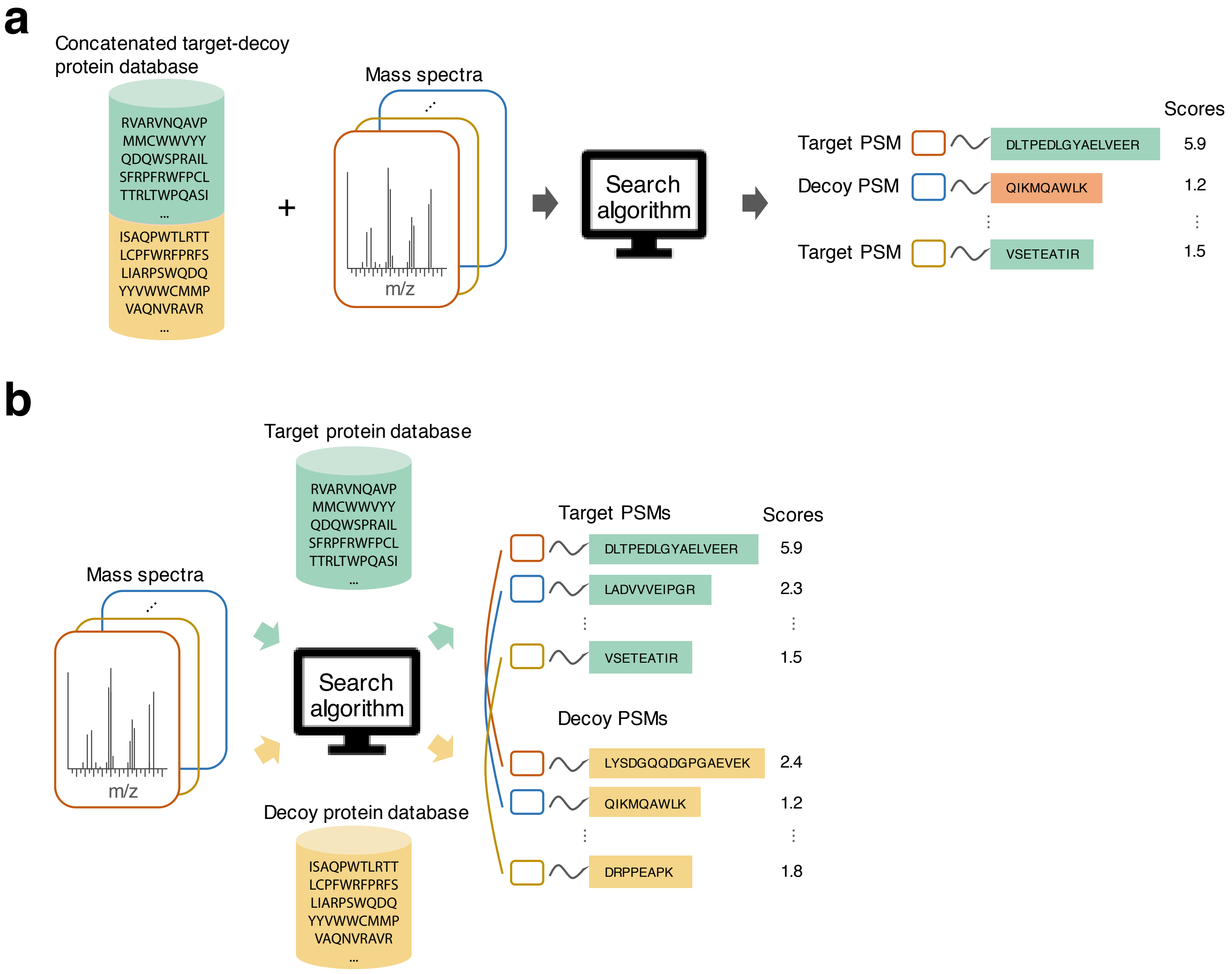

a
Concatenated target-decoy protein database
RVARVNQAVP
MMCWWVYY
QDQWSPRAIL
SFRPFRWFPCL
TTRLTWPQASI
...
ISAQPWTLRTT
LCPFWRFPRFS
LIARPSWQDQ
YYVWWCMMP
VAQNVRAVR
...
+
Mass spectra
m/z
Search algorithm
Scores
Target PSM
DLTPEDLGYAELVEER
5.9
Decoy PSM
QIKMQAWLK
1.2
Target PSM
VSETEATIR
1.5
b
Target protein database
RVARVNQAVP
MMCWWVYY
QDQWSPRAIL
SFRPFRWFPCL
TTRLTWPQASI
...
Mass spectra
m/z
Search algorithm
Decoy protein database
ISAQPWTLRTT
LCPFWRFPRFS
LIARPSWQDQ
YYVWWCMMP
VAQNVRAVR
...
Target PSMs
Scores
DLTPEDLGYAELVEER
5.9
LADVVVEIPGR
2.3
VSETEATIR
1.5
Decoy PSMs
LYSDGQQDGPGAEVEK
2.4
QIKMQAWLK
1.2
DRPPEAPK
1.8
