# Supplementary figures and images for: APIR: Aggregating Universal Proteomics Database Search Algorithms for Peptide Identification with FDR Control
Source: Genomics Proteomics Bioinformatics. 2024 Jun 3;22(2):qzae042. doi: 10.1093/gpbjnl/qzae042 (PMC12536914; doi:10.1093/gpbjnl/qzae042)

q-value/PEP

- True PSMs
- False PSMs

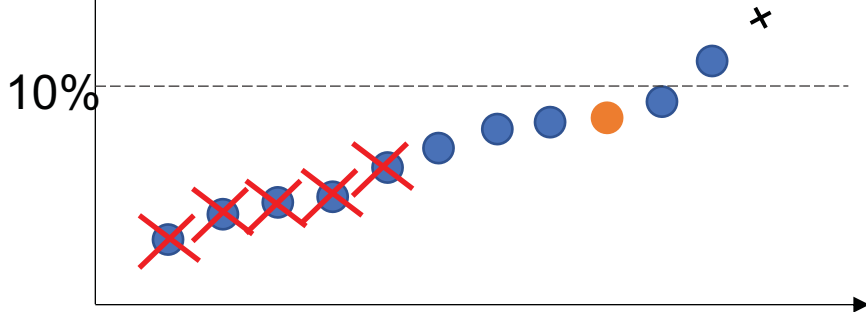

Supplement: qzae042_Supplementary_Data [file qzae042_supplementary_data.zip › Figure S4 E.pdf]

a

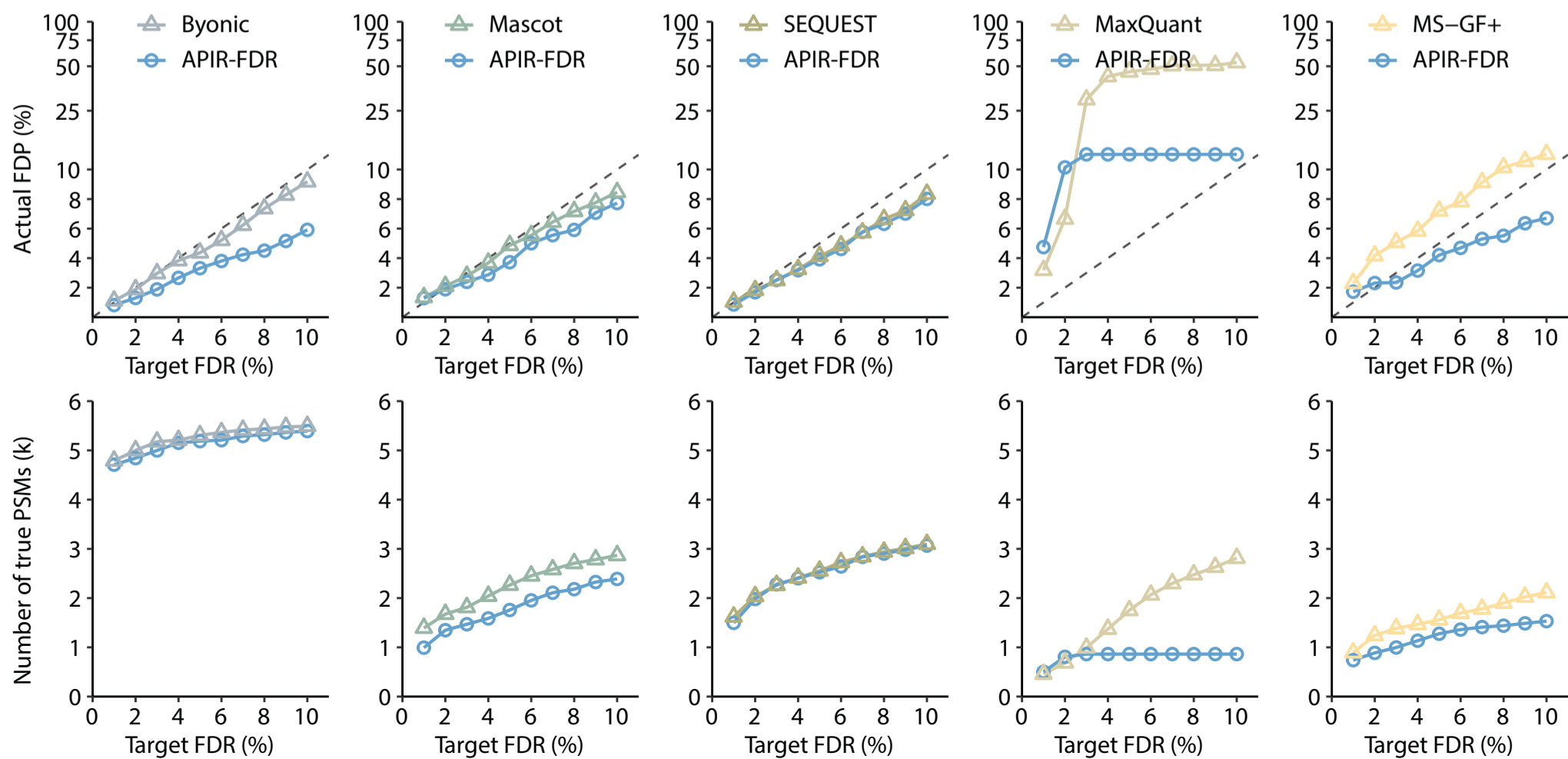

Supplement: qzae042_Supplementary_Data [file qzae042_supplementary_data.zip › Figure S5 E.pdf]

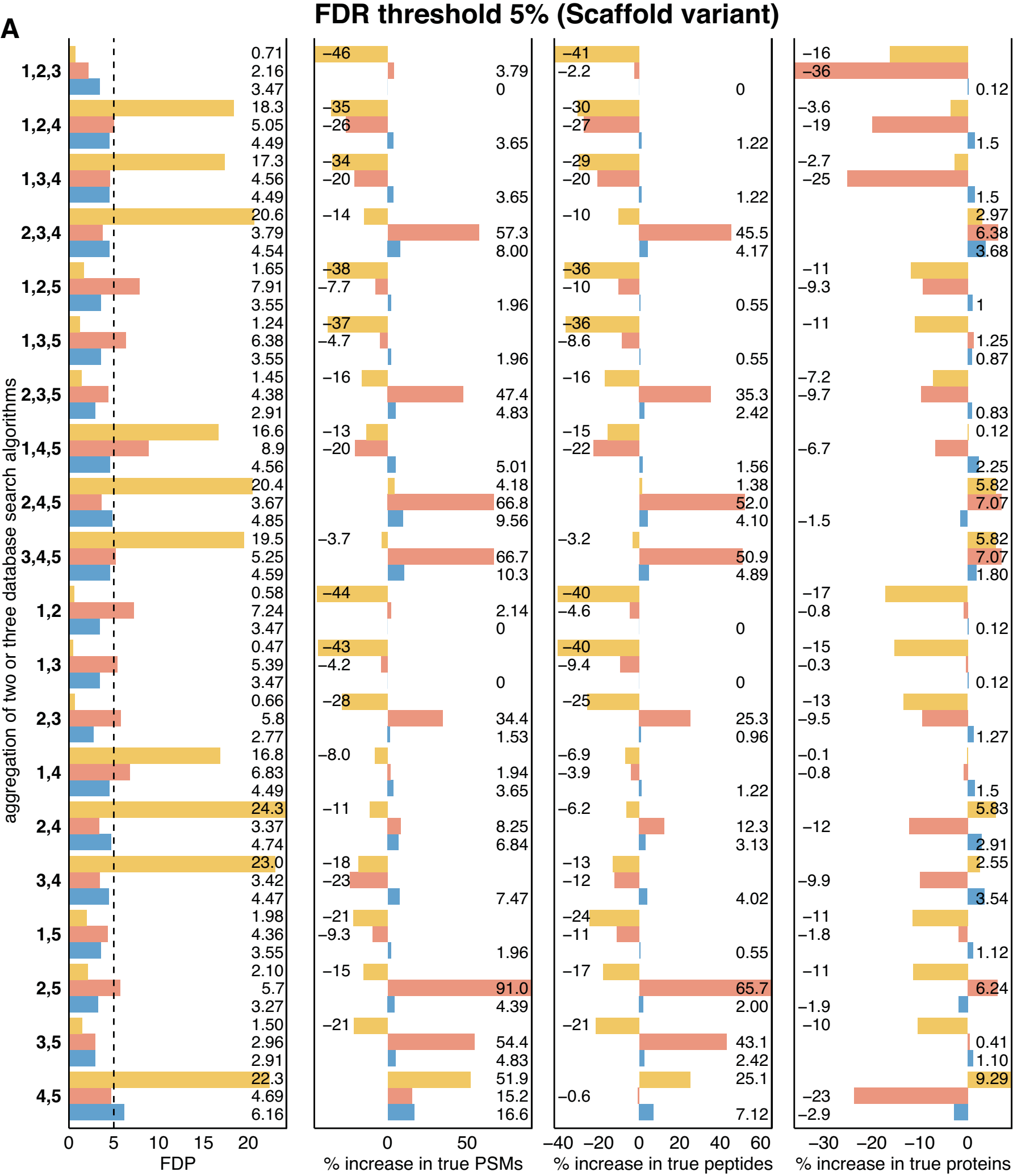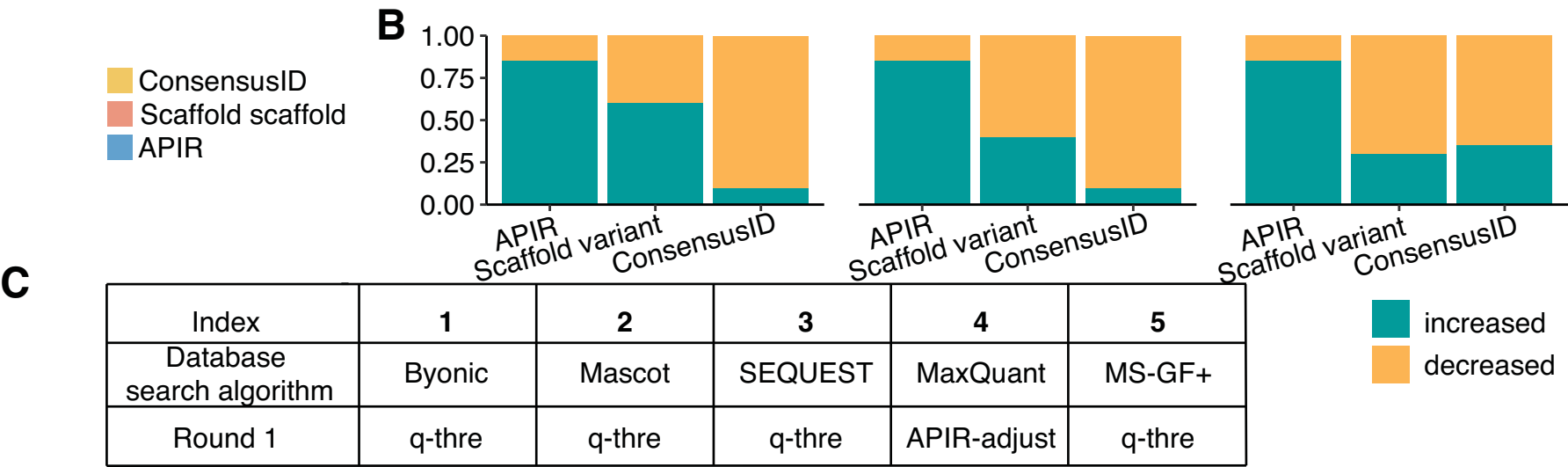

Supplement: qzae042_Supplementary_Data [file qzae042_supplementary_data.zip › Figure S7 E.pdf]

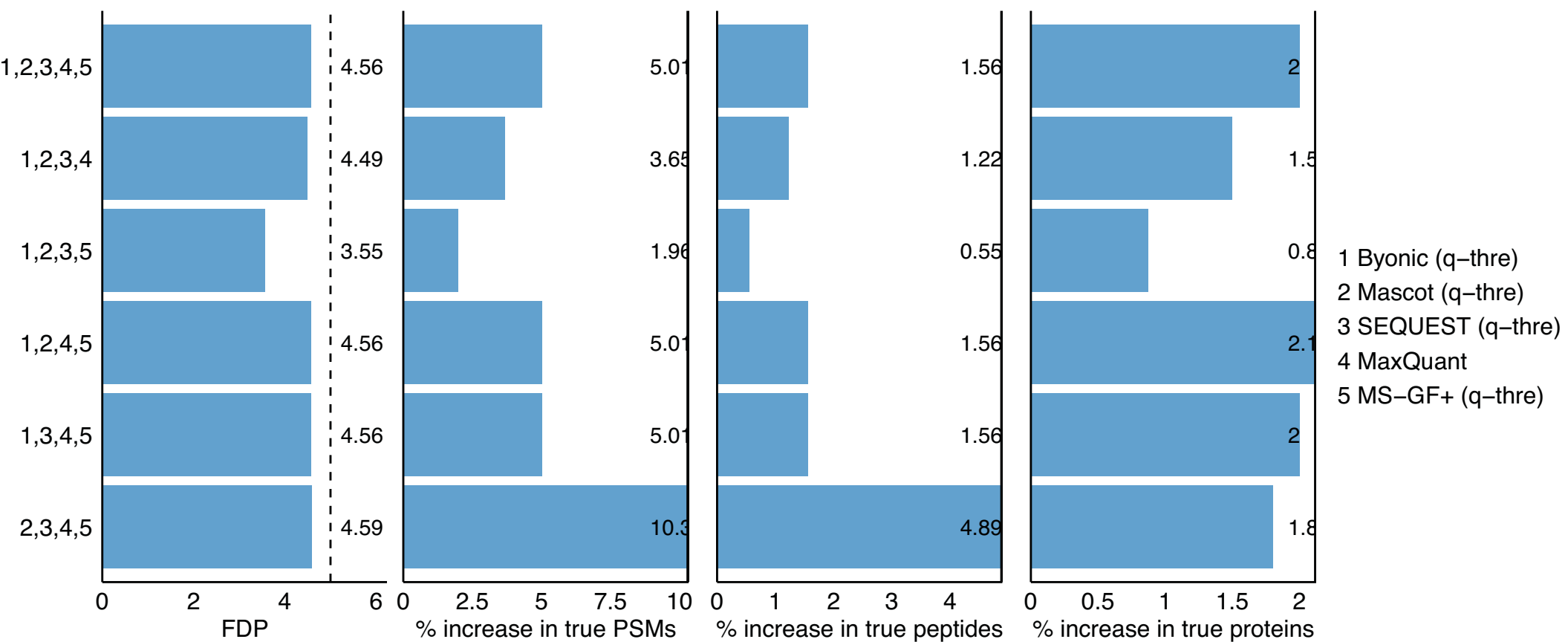

Supplement: qzae042_Supplementary_Data [file qzae042_supplementary_data.zip › Figure S9 E.pdf]

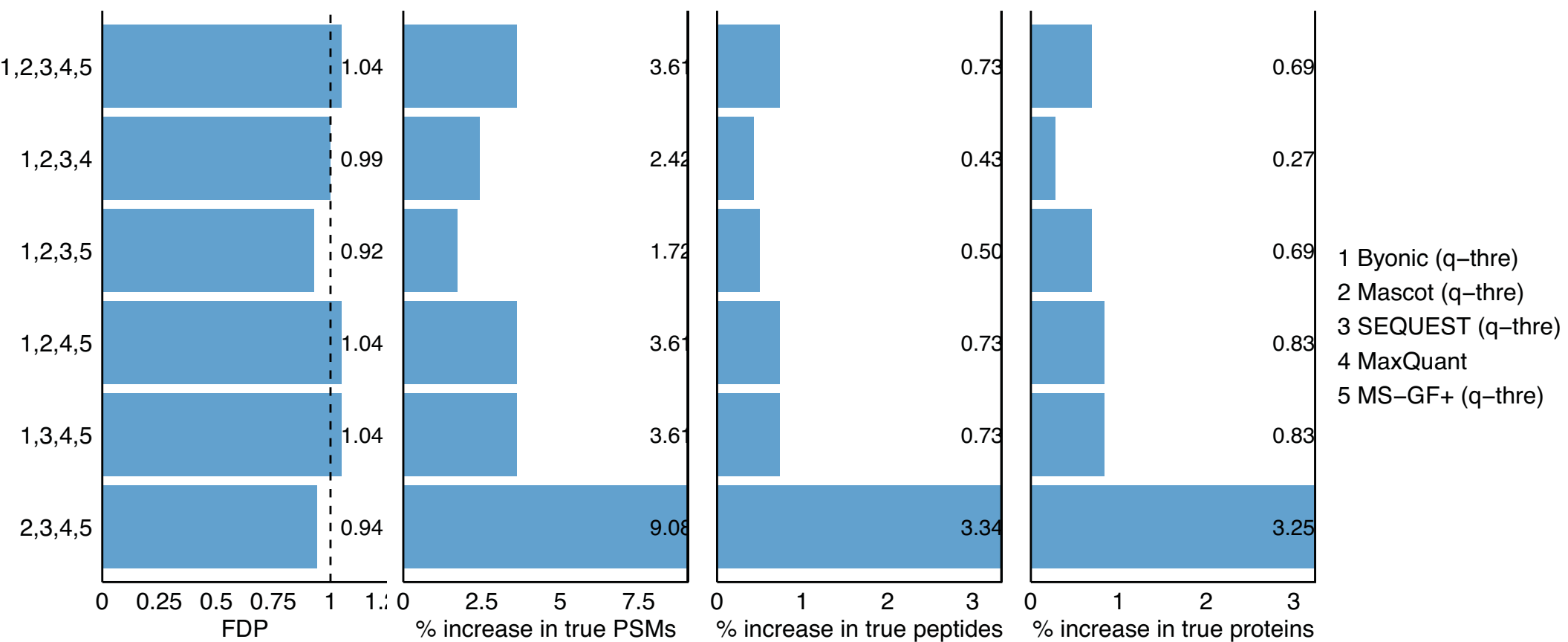

Supplement: qzae042_Supplementary_Data [file qzae042_supplementary_data.zip › Figure S10 E.pdf]

Target FDR = 1%

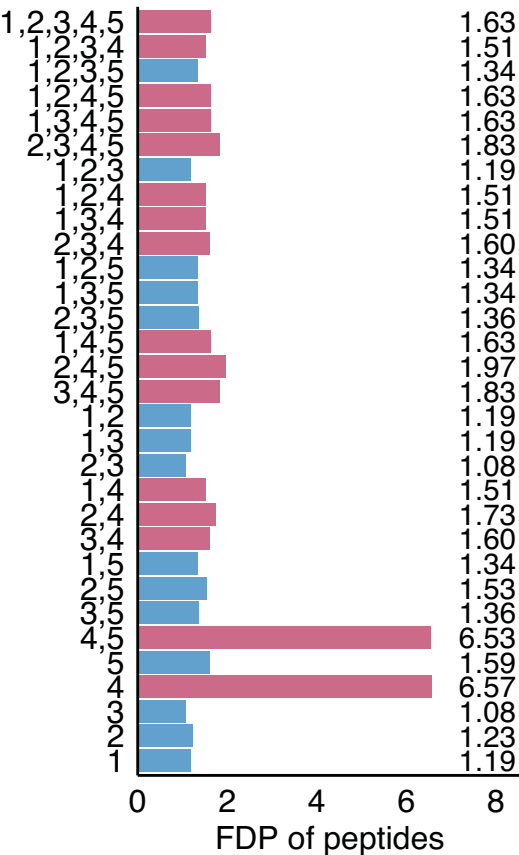

Target FDR = 5%

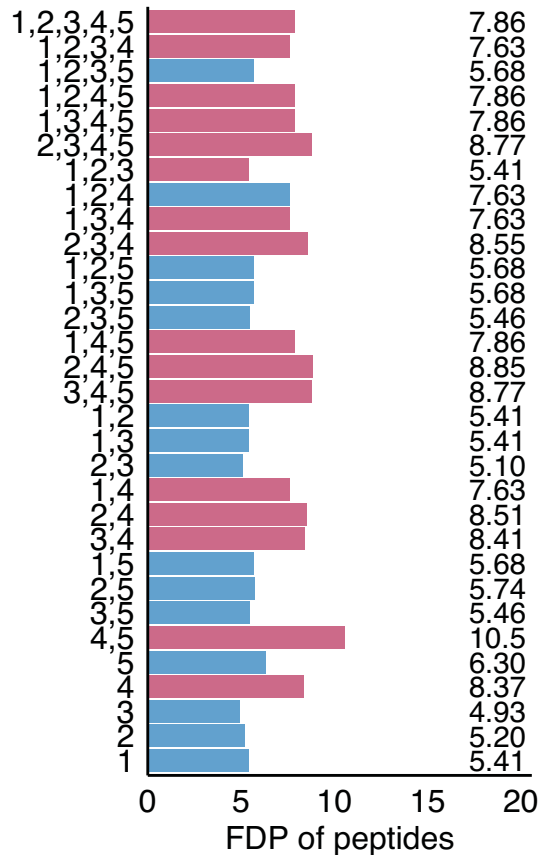

- 1 Byonic (q-thre)
- 2 Mascot (q-thre)
- 3 SEQUEST (q-thre)
- 4 MaxQuant
- 5 MS-GF+ (q-thre)

Supplement: qzae042_Supplementary_Data [file qzae042_supplementary_data.zip › Figure S11 E.pdf]

## A Phospho AML-C1

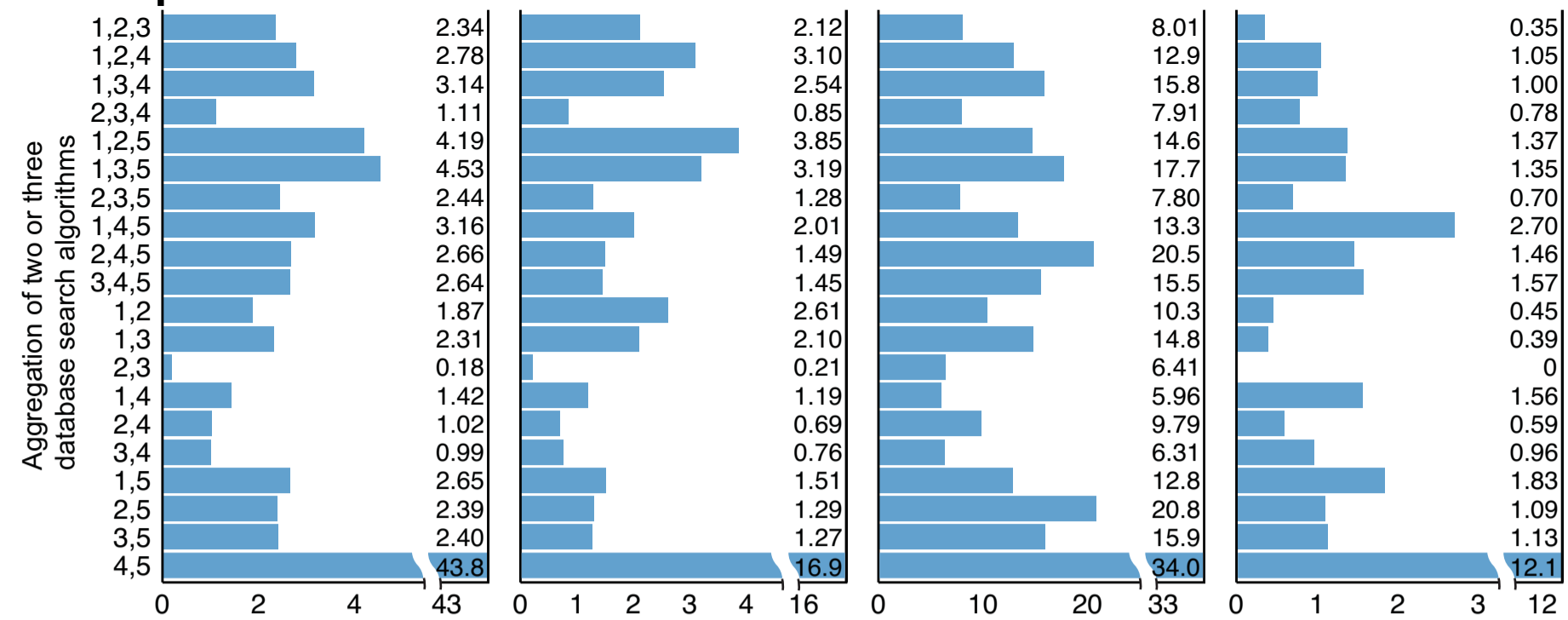

## B Phospho AML-C2

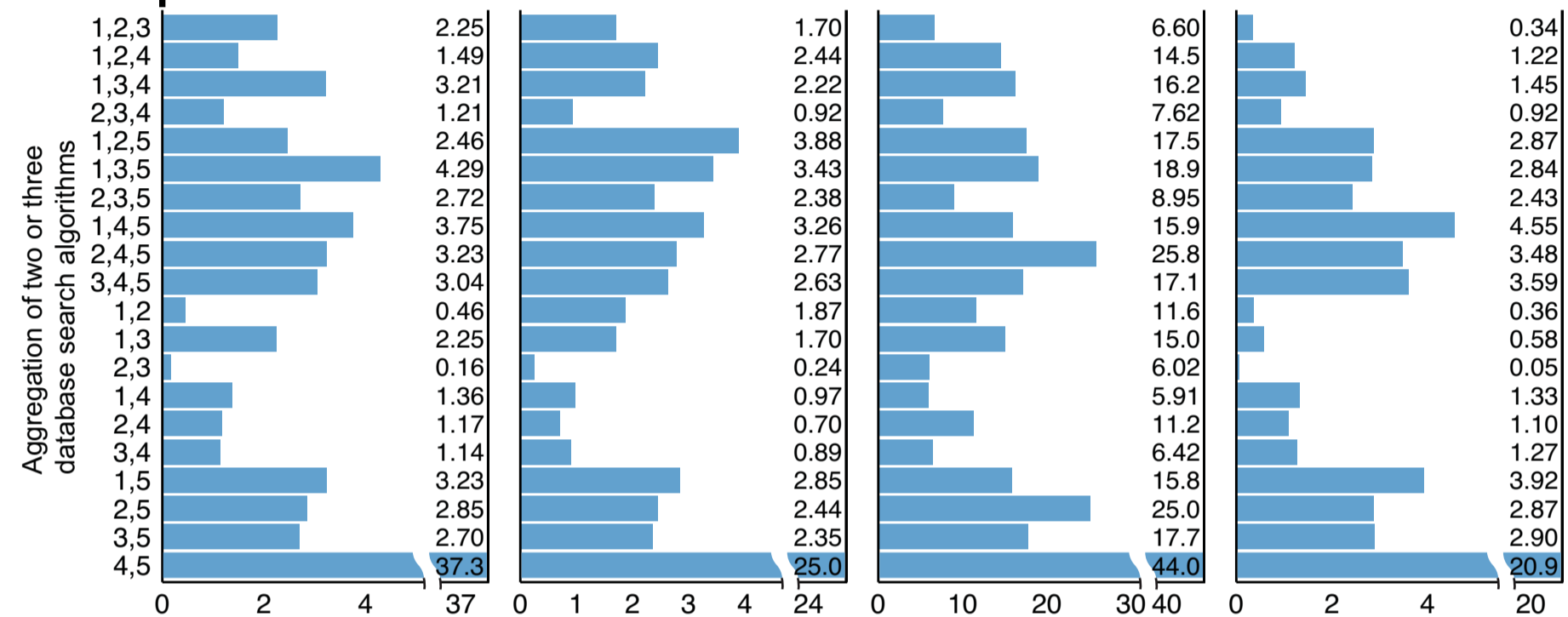

## C TNBC

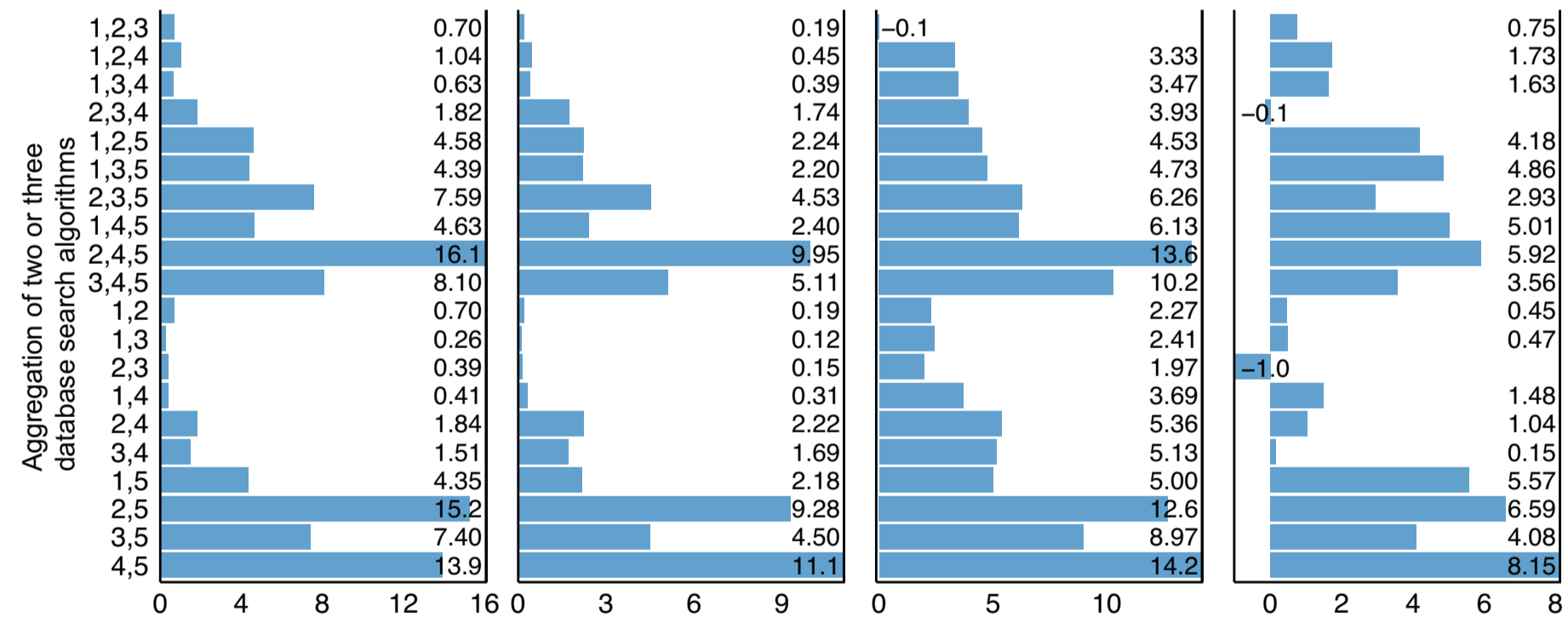

## D Nonphospho AML

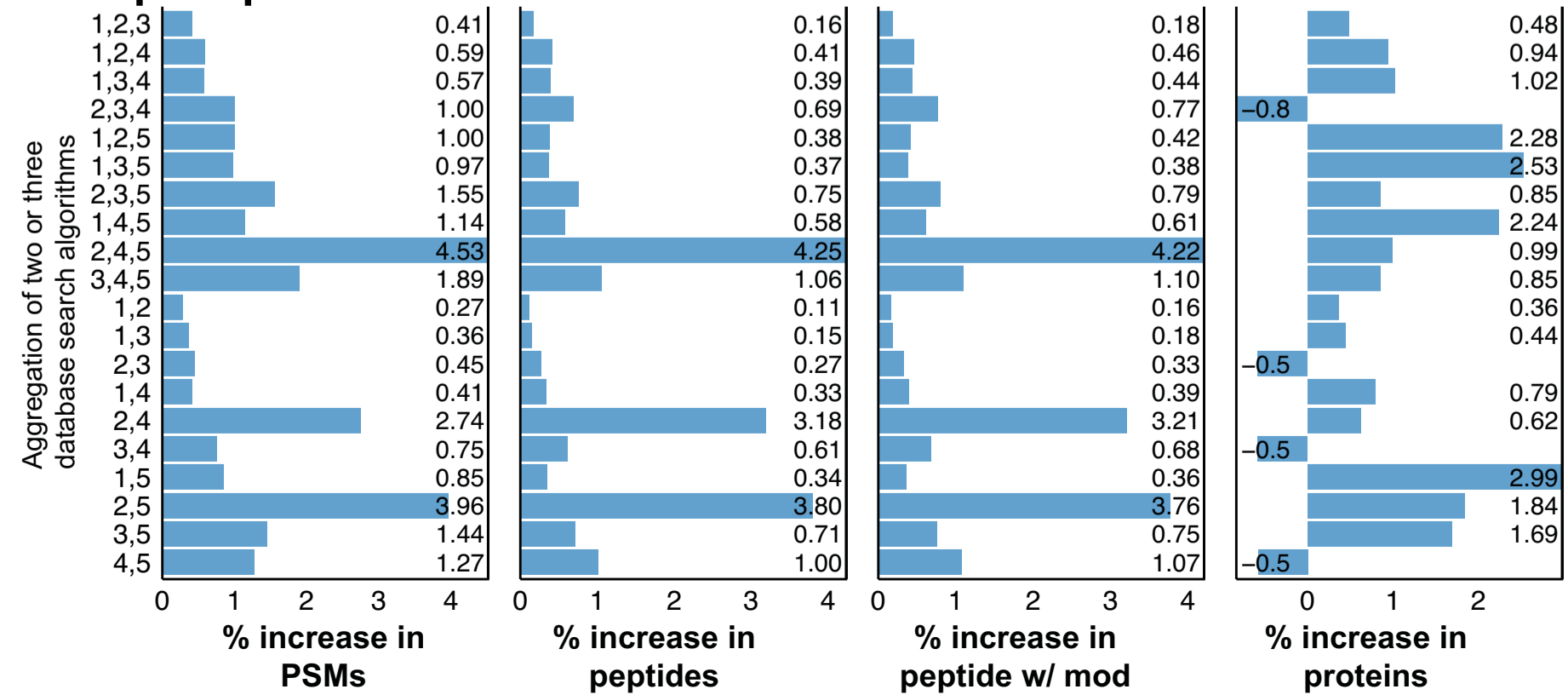

Supplement: qzae042_Supplementary_Data [file qzae042_supplementary_data.zip › Figure S12 E.pdf]

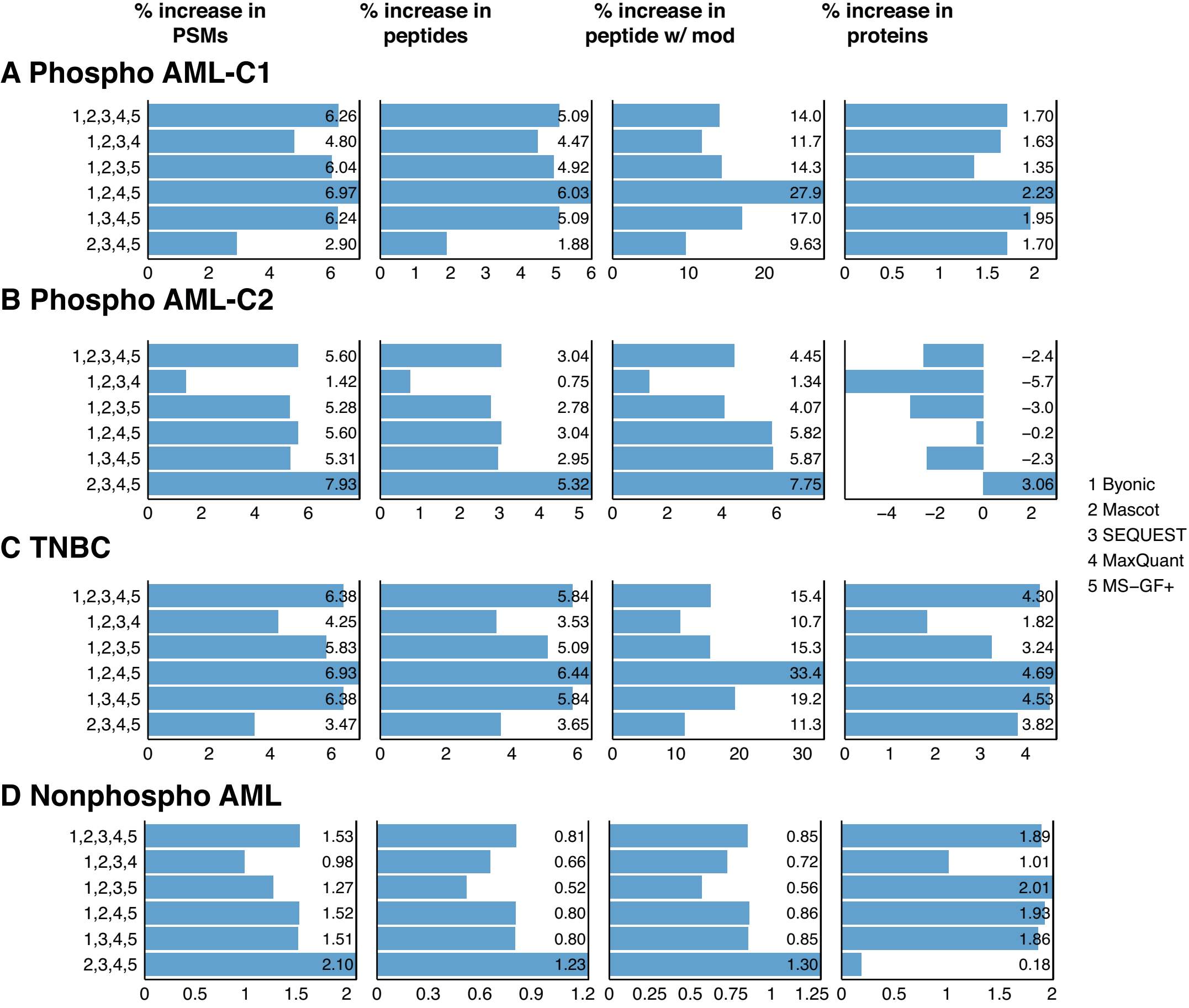

Supplement: qzae042_Supplementary_Data [file qzae042_supplementary_data.zip › Figure S13 E.pdf]

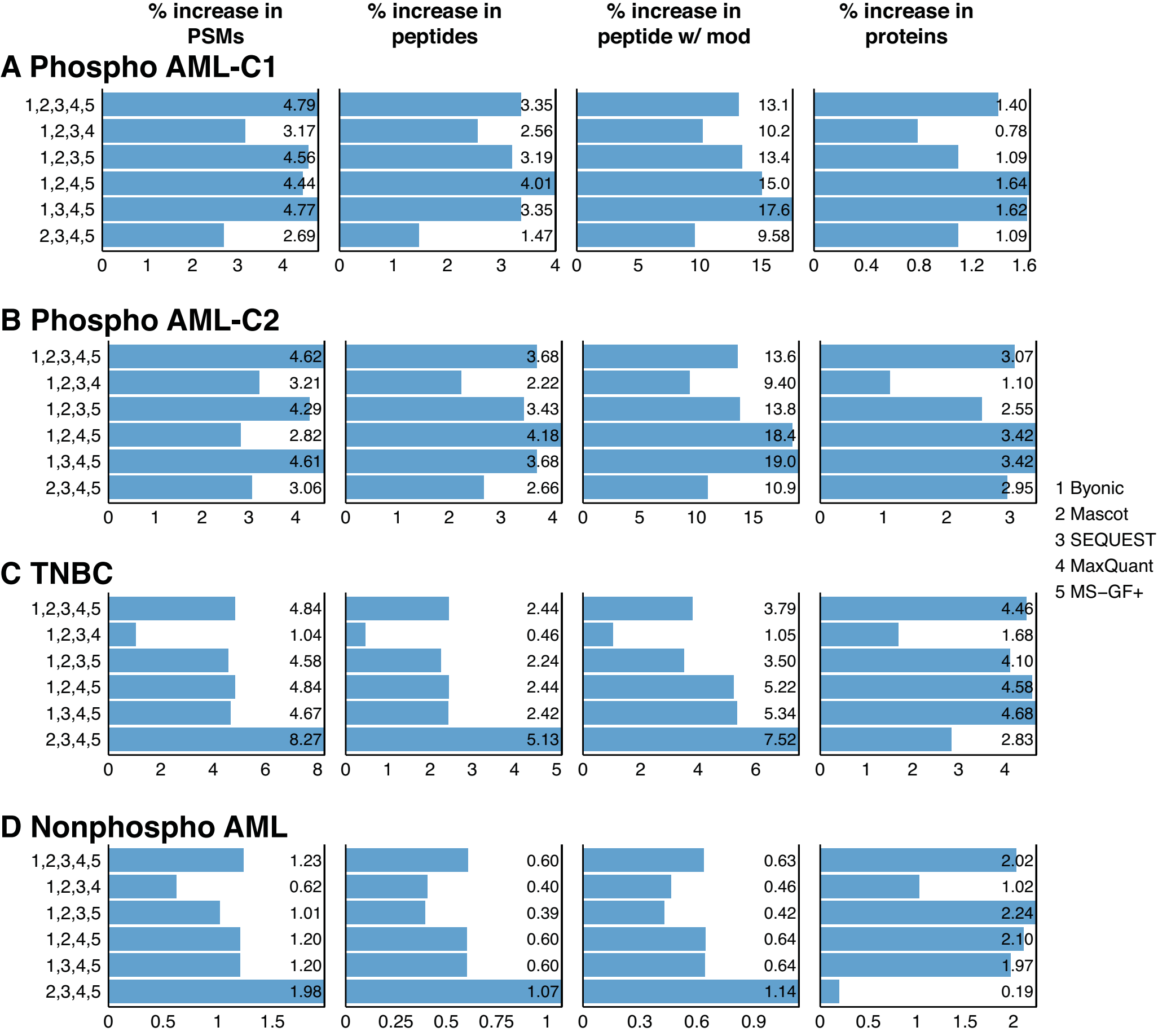

Supplement: qzae042_Supplementary_Data [file qzae042_supplementary_data.zip › Figure S14 E.pdf]

**FDR = 1%**

**FDR = 5%**

MaxQuant

MS-GF+

MaxQuant

MS-GF+

**Phospho  
AML-C1**

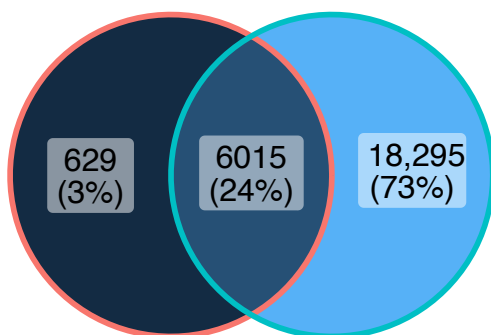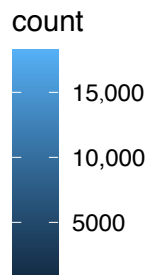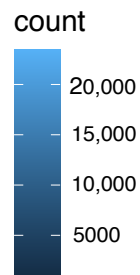

**Phospho  
AML-C2**

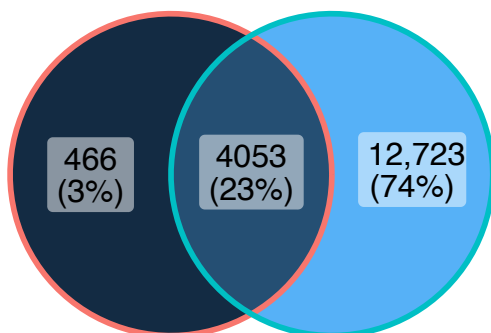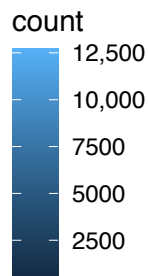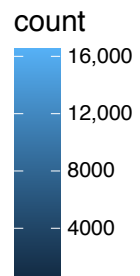

**TNBC**

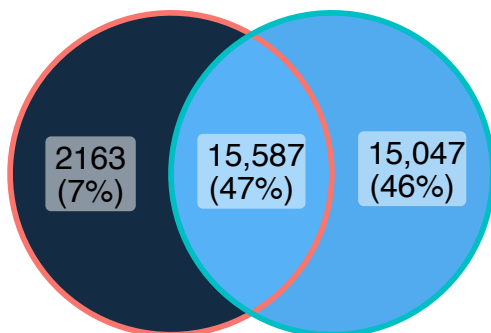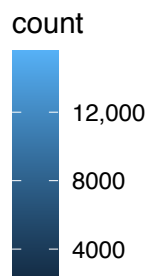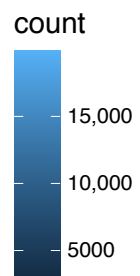

**Nonphospho**

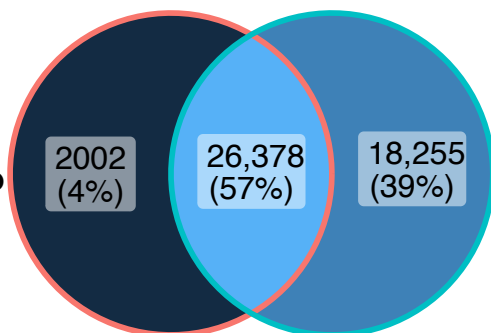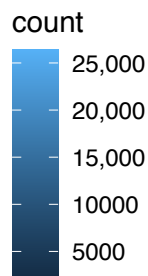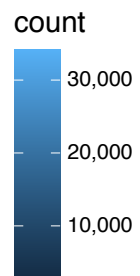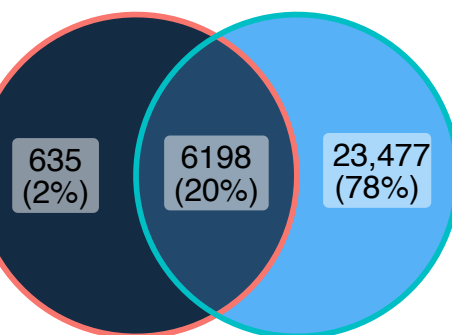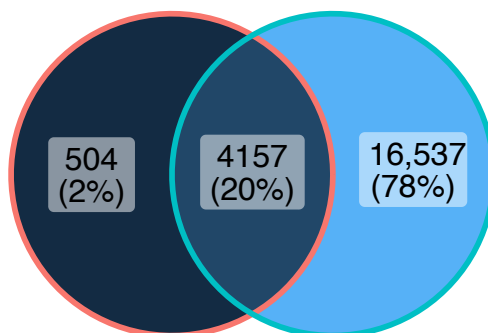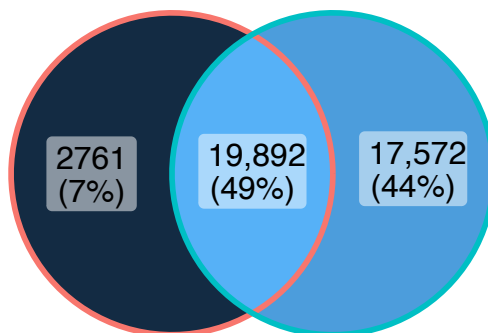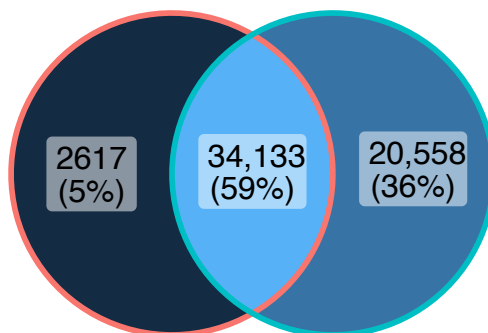

Supplement: qzae042_Supplementary_Data [file qzae042_supplementary_data.zip › Figure S15 E.pdf]

**True PSMs at target FDR = 1%**

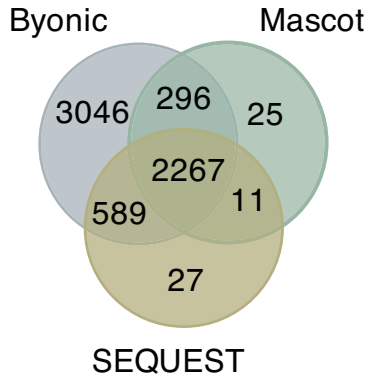

**True PSMs at target FDR = 5%**

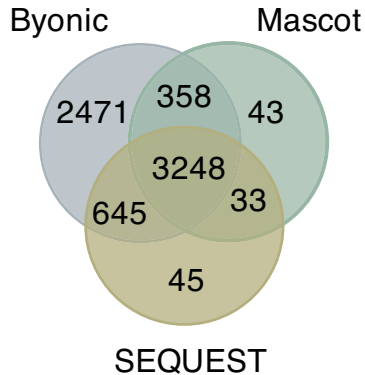

Supplement: qzae042_Supplementary_Data [file qzae042_supplementary_data.zip › Figure S2 E.pdf]

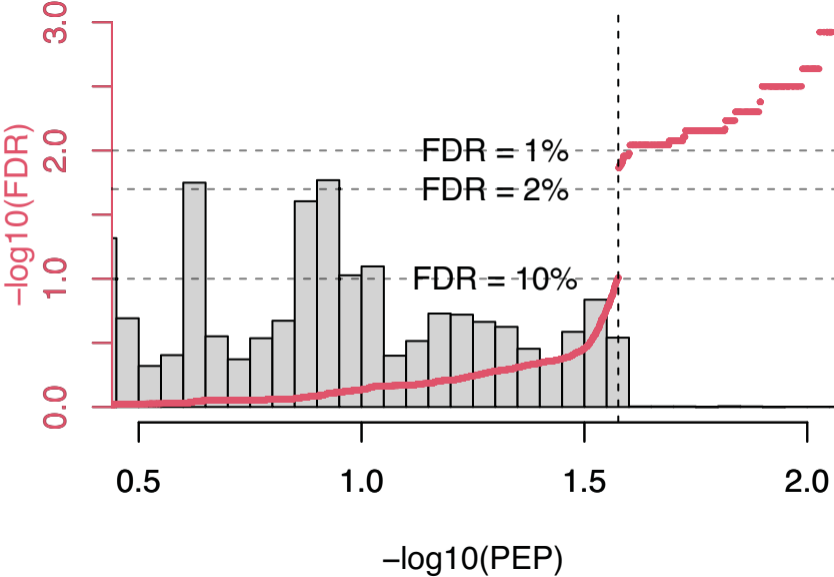

Supplement: qzae042_Supplementary_Data [file qzae042_supplementary_data.zip › Figure S3 E.pdf]
